# Onsite GTP fuelling via DYNAMO1 drives division of mitochondria and peroxisomes

**DOI:** 10.1038/s41467-018-07009-z

**Published:** 2018-11-06

**Authors:** Yuuta Imoto, Yuichi Abe, Masanori Honsho, Kanji Okumoto, Mio Ohnuma, Haruko Kuroiwa, Tsuneyoshi Kuroiwa, Yukio Fujiki

**Affiliations:** 1 Division of Organelle Homeostasis, Medical Institute of Bioregulation, Kyushu University, 3-1-1 Maidashi, Higashi-ku, Fukuoka 812-8582 Japan; 2 Department of Biology, Faculty of Sciences, Kyushu University, 744 Motooka Nishi-ku, Fukuoka, 819-0395 Japan; 3 Institute of Technology, Hiroshima College, 4272-1 Higashino, Osaki kamijima-cho, Toyota-gun, Hiroshima 725-0231 Japan; 4 Department of Chemical and Biological Science, Faculty of Science, Japan Women’s University, 2-8-1 Mejirodai, Bunkyo-ku, Tokyo 112-8681 Japan

## Abstract

Mitochondria and peroxisomes proliferate by division. During division, a part of their membrane is pinched off by constriction of the ring-shaped mitochondrial division (MD) and peroxisome-dividing (POD) machinery. This constriction is mediated by a dynamin-like GTPase Dnm1 that requires a large amount of GTP as an energy source. Here, via proteomics of the isolated division machinery, we show that the 17-kDa nucleoside diphosphate kinase-like protein, dynamin-based ring motive-force organizer 1 (DYNAMO1), locally generates GTP in MD and POD machineries. DYNAMO1 is widely conserved among eukaryotes and colocalizes with Dnm1 on the division machineries. DYNAMO1 converts ATP to GTP, and disruption of its activity impairs mitochondrial and peroxisomal fissions. DYNAMO1 forms a ring-shaped complex with Dnm1 and increases the magnitude of the constricting force. Our results identify DYNAMO1 as an essential component of MD and POD machineries, suggesting that local GTP generation in Dnm1-based machinery regulates motive force for membrane severance.

## Abstract (web summary)

Mitochondria and peroxisomes require a dynamin-like GTPase to remodel membranes during division. Here the authors identify DYNAMO1, a nucleoside diphosphate kinase-like protein that generates a local source of GTP to promote constriction of the division machinery and produce daughter organelles.

## Introduction

Membrane fission is essential for the life of cells. During cell division, the cell membrane is cleaved at the equatorial plane. Cellular communication from hormone signaling to neurotransmission is supported by a process known as endocytosis. For proliferation of intracellular organelles such as mitochondria and peroxisomes, portions of their membrane are severed to generate daughter organelles. These fission reactions are essential for all intracellular membrane remodeling events and are mediated by the dynamin family of GTPase proteins^1^. These proteins polymerize and form a ring or spiral structure to constrict and pinch off the membrane^2^. Typical dynamin family members are Dnm1, which mediates division of mitochondria^3^ and peroxisomes^4^, and dynamin isoforms that pinch off the neck of endocytic pits^5^. They are among the most powerful motor proteins and are capable of constricting membrane gaps as large as a few hundred nanometers in less than 1 min^6,7^. Because the diameters of mitochondrial and peroxisomal division planes are substantially larger than the neck of an endocytic pit, the magnitude of the constriction of in vitro Dnm1 (~50 nm) is fivefold higher than that of dynamin (~10 nm)^7^. Despite the high magnitude of its constriction, Dnm1 has a weak affinity for GTP and relatively high rate of GTP hydrolysis^8^ as similar to dynamin^9^. Furthermore, high rate of GTPase activity is enhanced by polymerization (*k*_cat_ = 50 min^−1^)^8^ much higher than that of small GTPase (*k*_cat_ = ∼8 × 10^−3^ min^−1^)^10^. The fact that significant constriction mediated by Dnm1 requires high levels of GTP (~1 mM)^7^, indicates that efficient replenishment of GTP is needed to sustain the activity of assembly and constriction. However, how the functions of Dnm1 are energetically supported is unclear. Accessory proteins of Dnm1, such as Fis1, Mdv1, Mff, and MiDs, might enhance the kinetics of its GTP affinity and facilitate membrane constriction^11^. However, conservation of these proteins among eukaryotes is limited, even though Dnm1-mediated division of mitochondria and peroxisomes is common to almost all eukaryotic cells including those in animals, fungi, land plants, and algae^12^.

To search for a fundamental protein that potentially functions in energetic regulation and kinetics of Dnm1, proteomic analysis of the division machinery is required. For this approach, isolation of the division machineries is essential. However, most of the eukaryotic cells contain numerous numbers of mitochondria and peroxisomes that divide randomly, which makes it difficult to collect bulk of intermediate of dividing organelles. In contrast, a unicellular red alga, *Cyanidioschyzon merolae* (*C. merolae*), has advantages to conquer this problem because it contains only one peroxisome, mitochondrion, and plastid per cell. Moreover, division of these organelles can be highly synchronized by light/dark stimulation in *C. merolae* (Supplementary Figure 1)^13,14^. This feature enables bulk isolation of mitochondrial and peroxisomal division machineries^15–17^. The division machinery of mitochondria, called mitochondrial division (MD) machinery, has a ring-shaped electron-dense structure with a diameter of 150–1200 nm^12^. This machinery consists of an outer ring formed around the cytoplasmic side of the mitochondrial outer membrane and an inner ring formed around the matrix side of the inner membrane. The outer ring is a dynamin-based ring containing Dnm1 and skeletal polyglucan filaments, called the MD ring^16,18,19^. The inner ring is a remnant of a bacterial cell division apparatus containing FtsZ^20^ that *Opisthokonta* have lost^21,22^. However, MD machinery is physically associated with plastid dividing (PD) machinery, implying that the isolated MD machinery fraction may be contaminated with plastid division-associated proteins^15,16,23^. In contrast, the division machinery of peroxisomes is formed at different times and sites than MD and PD machineries^17,24^. The peroxisome-dividing (POD) machinery with a diameter of 50–600 nm is composed of dynamin-based (DB) rings and skeletal filamentous rings formed at the cytoplasmic side of peroxisomal membranes^17^. The DB ring is structurally analogous to the outer ring of MD machinery and also contains Dnm1^17^. Moreover, the DB ring can be physically separated from the other components of POD machinery, which enables mapping of candidate proteins to specific structures.

By using the unicellular red alga, *C. merolae*, 17-kDa nucleoside diphosphate kinase-like protein, dynamin-based ring motive-force organizer 1 (DYNAMO1) is identified as a fundamental component of MD and POD machineries. DYNAMO1 directly binds to Dnm1 and localizes on the Dnm1-based ring structure in the machineries. DYNAMO1 generates GTP from ATP and GDP and this enzyme activity is essential to sustain Dnm1-mediated fission of mitochondrion and peroxisome. Furthermore, in vitro analysis demonstrates that DYNAMO1 enhances constriction force generated by Dnm1-based rings. Thus, energy source for the constriction of MD and POD machineries is most likely generated locally around the membrane scission sites during the division of mitochondrion and peroxisome.

## Results

### Identification of DYNAMO1 from isolated POD machinery

**Fig. 1:**
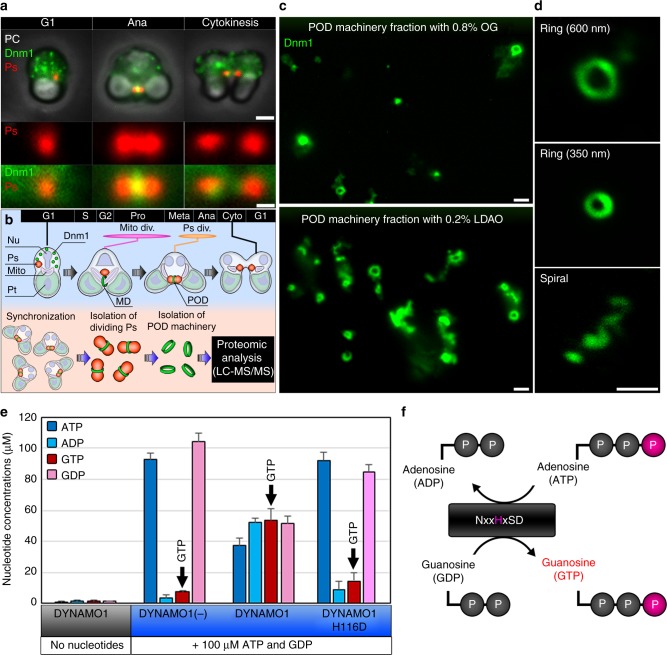
Proteomic analysis of POD machinery and identification of DYNAMO1. **a** Phase contrast (PC) and immunofluorescence images of *C. merolae* cells during G1 phase, anaphase, and cytokinesis. Ps peroxisome (anti-catalase antibody), Dnm1 (anti-Dnm1 antibody). **b** Schematic representing isolation and proteomic analysis of POD machinery. Nu cell nucleus, Mito mitochondrion, Pt plastid, Mito div. mitochondrial division period, Ps div peroxisomal division period. **c** Upper panel shows the 0.8% OG-treated POD machinery fraction and the lower panel shows the 0.2% LDAO-treated POD machinery fraction. **d** Typical structures of isolated POD machinery stained with the anti-Dnm1 antibody. **e** LC–ESI–MS/MS analysis of the nucleoside diphosphate kinase activity of recombinant DYNAMO1. **f** Schematic representing a working model of nucleoside diphosphate kinase. Data in **e** are means ± s.d. (*n* = 3). Scale bars: 1 μm (**a**, upper panels); 500 nm (**a**, lower panels; **c**, **d**)

**Fig. 2:**
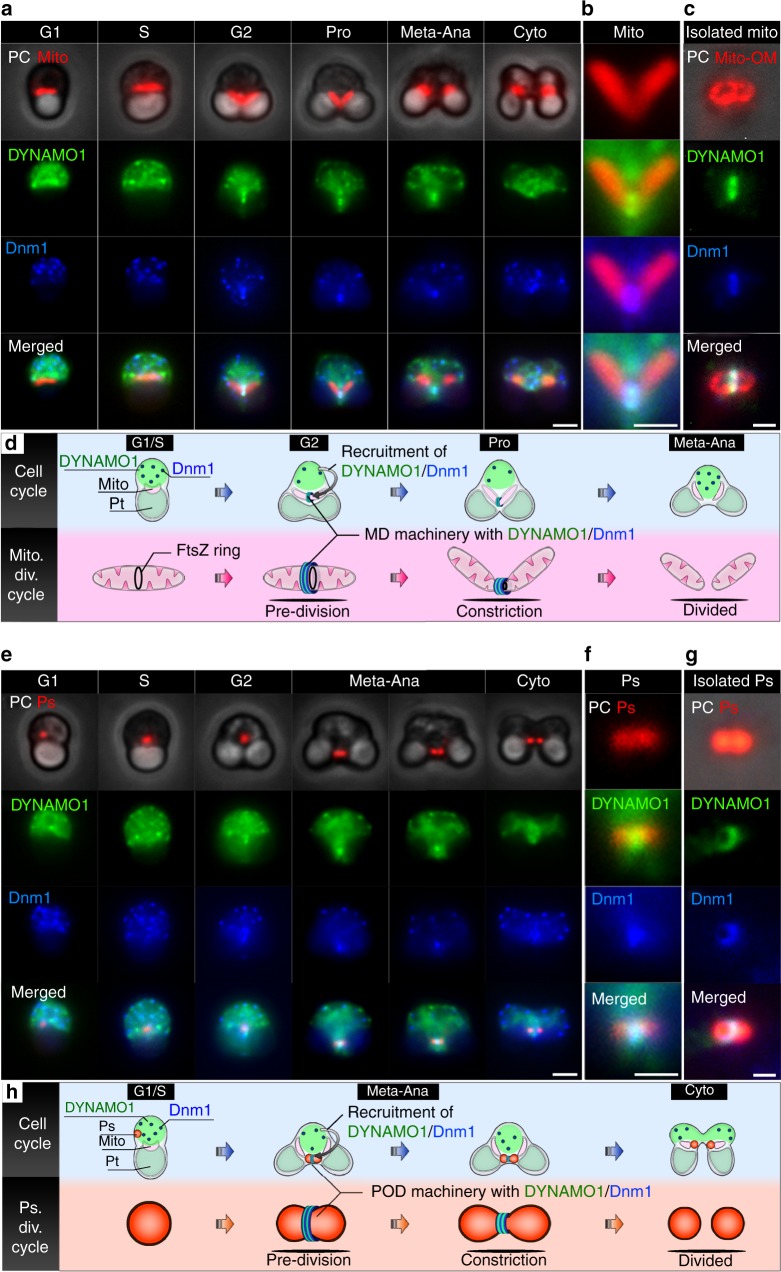
Dynamics of DYNAMO1 during the division periods of mitochondria and peroxisomes. **a** Phase contrast and immunofluorescence microscopy images of a mitochondrion (Mito, anti-EF-Tu antibody), DYNAMO1 (anti-DYNAMO1 antibody), and Dnm1 (anti-Dnm1 antibody) during the indicated cell cycle phases. Pro prophase, Meta–Ana metaphase–anaphase, Cyto cytokinesis. **b** Magnified image around the mitochondrial division site during prophase shown in **a**. **c** Localization of DYNAMO1 and Dnm1 on the isolated mitochondrial outer membrane [Mito-OM, anti-porin (POR) antibody]. **d** Schematic representing DYNAMO1 dynamics during the mitochondrial division period. **e** Phase contrast and immunofluorescence microscopy images of a peroxisome (Ps), DYNAMO1, and Dnm1 during the indicated cell cycle phases. **f** Magnified image around the peroxisomal division site during meta-anaphase shown in **e**. **g** Localization of DYNAMO1 and Dnm1 on an isolated dividing peroxisome. **h** Schematic representing DYNAMO1 dynamics during the peroxisomal division period. Scale bars: 1 μm (**a**, **e**); 500 nm (**b**, **c**, **f**, and **g**)

We investigated the molecular identity of Dnm1-based machinery by proteomic analysis of the POD machinery-enriched fraction (Fig. 1a, b). To prepare sufficient amounts of POD machinery, we improved existing isolation methods, including treatment with 0.2% lauryldimethylamine-N-oxide (LDAO) instead of 0.8% *n*-octyl-β-d-glucoside (OG)^17,25^, to greatly increase the yield of Dnm1-positive rings (Fig. 1c, d; Supplementary Figure 2a and b). The LDAO-treated fraction of POD machinery contained spiral structures (Fig. 2d) similar to those formed by Dnml GTPase in MD machinery^15^. Thus, the LDAO-treated fraction of POD machinery possibly contained intact POD machinery with retention of its in vivo functions. To identify components of the DB ring, the solubilized fraction of isolated POD machinery was compared with the interphase fraction. Six bands distinctly enriched in the POD machinery fraction were analyzed by liquid chromatography and mass spectrometry (LC–MS/MS) and peptide mass fingerprinting using the complete genomic sequence of *C. merolae*^14,26^. Dnm1 was evidently identified in band 1 at ~90 kDa (Supplementary Figure 2c; Supplementary Table 1), indicating enrichment of POD machinery. Among the detected candidate proteins, we found that the gene product of *CML110C*, a top-assigned protein in the most enriched band 5 (Supplementary Figure 2c; Supplementary Table 1), bound to Dnm1 in vitro and in vivo (Supplementary Figure 2d–g), suggesting that CML110C is a novel 17 kDa component of the DB ring. CML110C, termed as DYNAMO1, contains a consensus motif of nucleoside diphosphate kinase (NDPK) (Supplementary Figure 2h) that forms various oligomeric structures, such as a hexamer, tetramer, and dimer, and catalyzes conversion of ATP to GTP^27–29^. Recombinant DYNAMO1 formed a tetramer (Supplementary Figure 2i–k) and generated GTP upon addition of ATP plus GDP (Fig. 1e, f). When the histidine residue at position 116 of DYNAMO1 in the consensus active site motif was replaced by aspartic acid (DYNAMO1 H116D), its GTP generation was abolished (Fig. 1e), demonstrating that DYNAMO1 indeed functions as a nucleoside diphosphate kinase. Because nucleoside diphosphate kinase is involved in dynamin-mediated endocytosis and OPA1-mediated mitochondrial fusion in mammalian cells^29,30^, we suggest that DYNAMO1 is involved in Dnm1-based organelle division machineries.

### Localization of DYNAMO1 at the organelle division sites

To investigate whether DYNAMO1 is involved in organelle division, we immunocytochemically analyzed endogenous DYNAMO1 with an anti-DYNAMO1 antibody. DYNAMO1 was expressed continuously during the cell cycle and was detected in both fractions of organelles containing peroxisomes and mitochondria and cytosol (Supplementary Figure 3a and b). Notably, 15–25% of DYNAMO1 were detected in organelle fractions as compared to cytosolic fraction at the G2–M cell cycle phase, when mitochondria and peroxisomes divide (Supplementary Figure 3b-d). Therefore, DYNAMO1 is likely involved in not only peroxisomal, but also mitochondrial division. We next analyzed localization of DYNAMO1 and Dnm1 at each cell cycle phase (Fig. 2). DYNAMO1 was initially distributed in the cytosol during G1 phase and then partially accumulated at both edges of the mitochondrion during S phase when the inner FtsZ ring formed at the destined division site of each mitochondrion (Supplementary Figure 4a and b). Subsequently, DYNAMO1 was recruited to Dnm1-labeled division sites of mitochondria during G2–prophase (Fig. 2a, b). Immediately after mitochondrial division, puncta of DYNAMO1 and Dnm1 apparently disappeared from the mitochondria. Furthermore, DYNAMO1 was almost completely colocalized with Dnm1 at the division site and across the mitochondrial outer membrane of isolated mitochondria (Fig. 2c; Supplementary Figure 4c). Therefore, the dynamics of DYNAMO1 were cooperative with Dnm1 as a likely component of the outer ring of MD machinery (Fig. 2d). After mitochondrial division, DYNAMO1 was localized to the division sites of peroxisomes only during metaphase–anaphase and those of isolated peroxisomes (Fig. 2e–h; Supplementary Figure 4d). After peroxisome division, DYNAMO1 and Dnm1 signals were absent in peroxisomes, suggesting that DYNAMO1 is a component of POD machinery.

### DYNAMO1 is a basic component of MD and POD machinery

**Fig. 3:**
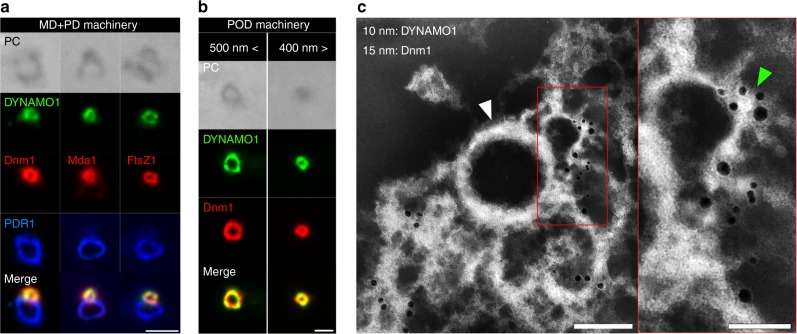
Localization of DYNAMO1 in MD and POD machineries. **a** Phase contrast and immunofluorescence microscopy images of DYNAMO1, Dnm1, Mda1 (anti-Mda1 antibody), FtsZ1 (anti-FtsZ1-antibody), and PDR1 (marker protein for PD machinery, anti-PDR1-antibody) on an isolated MD/PD machinery complex. **b** Phase contrast and immunofluorescence microscopy images of DYNAMO1 and Dnm1 on isolated POD machineries with diameters of >500 and <400 nm. **c** Whole-mount negative-staining immunoelectron microscopy images of DYNAMO1 (10 nm immunogold particles) and Dnm1 (15 nm immunogold particles) on isolated POD machinery. Right panel shows a magnified image of the red-boxed area in the left panel. The white arrowhead indicates a filamentous ring serving as a skeletal structure of POD machinery. The green arrowhead indicates a DB ring generating motive force for constriction. Scale bars: 1 μm (**a**); 500 nm (**b**); 200 nm (**c**); 50 nm (magnified image in **c**)

To confirm that DYNAMO1 is a component of MD and POD machineries, we investigated its localization in preparations of the isolated machineries (Fig. 3a–c; Supplementary Figure 5a). DYNAMO1 was colocalized with Dnm1 in MD machinery labeled with Mda1 and FtsZ, but not in PD machinery labeled with the PD ring component PDR1 (Fig. 3a). The DYNAMO1 and Dnm1 signal intensities in MD machinery were constant, regardless of the diameter of the ring (Supplementary Figure 5b), suggesting that DYNAMO1 is a fundamental component of MD machinery and functions together with Dnm1 during its entire constriction process. In the isolated POD machinery, DYNAMO1 was also colocalized with Dnm1, and the DYNAMO1 and Dnm1 signal intensities were constant, regardless of the diameter of the POD ring (Fig. 3b; Supplementary Figure 5c). We further explored nanoscale localization of DYNAMO1 by whole-mount negative-staining immunoelectron microscopy (Fig. 3c). The ultrastructure of the DB ring was defined by a Dnm1-labeled curved amorphous string that was partially peeled away from a rigid ring-shaped skeletal filamentous ring upon treatment with a nonionic detergent^17^. DYNAMO1-labeled 10 nm immunogold particles were detected on the string of the DB ring, but not on the skeletal filamentous ring, indicating that DYNAMO1 localizes on the DB ring formed at the cytosolic side of the division plane. These results strongly suggest that DYNAMO1 is a component of MD and POD machineries and generates GTP on their respective Dnm1-based rings.

### GTP-generating reaction of DYNAMO1 induces membrane fission

**Fig. 4:**
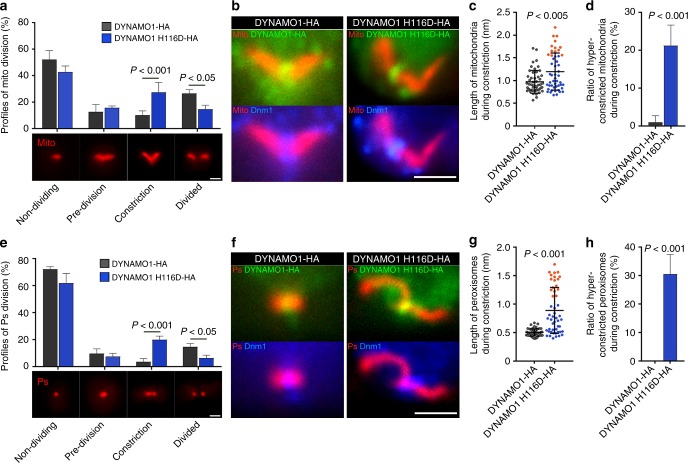
Disruption of DYNAMO1 functions and whole-mount negative-staining electron microscopy analysis of DYNAMO1-Dnm1 dynamics. **a** Profiles of mitochondrial division based on morphological changes during division. Mitochondria are labeled as in Fig. 2a. *n* = 3, at least 50 cells were counted in each experiment. **b** Localization of expressed DYNAMO1-HA and DYNAMO1 H116D-HA (Alexa 488-conjugated anti-HA antibody), Dnm1, and mitochondria in metaphase cells. **c** Length of mitochondria in cells expressing DYNAMO1-HA or DYNAMO1 H116D-HA. *n* = 50 in each experiment. Orange dots indicate hyper-constricted mitochondria as determined by quantification of their length that was elongated by more than 1.5 μm, which were positive for DYNAMO1 and Dnm1. **d** Ratio of hyper-constricted mitochondria during the constriction phase. **e** Profiles of peroxisomal division based on morphological changes during division. *n* = 3, at least 50 cells were counted in each experiment. **f** Localization of DYNAMO1-HA, DYNAMO1 H116D-HA, Dnm1, and peroxisomes in anaphase cells. **g** Lengths of peroxisomes in cells expressing DYNAMO1-HA or DYNAMO1 H116D-HA. *n* = 50 in each experiment. Orange dots indicate hyper-constricted peroxisomes as determined in **c** by quantification of their length that was elongated by more than 1 μm, which were positive for DYNAMO1 and Dnm1. **h** Ratio of hyper-constricted peroxisomes during the constriction phase. Scale bars: 1 μm. Data are means ± s.d. *P*; *p*-value (Mann–Whitney test)

To understand molecular function of DYNAMO1 on the MD and POD machinery, genetic disruption of DYNAMO1, particularly abolishment of nucleoside diphosphate kinase activity is needed. Nucleotide diphosphate kinases form an oligomer, including DYNAMO1 (Supplementary Figure 2j and k), and various dominant negative forms have been reported^31–33^. Thus, we next suppressed DYNAMO1 functions by transient expression of DYNAMO1 H116D, a DYNAMO1 mutant defective for GTP-generating activity (Fig. 1e). DYNAMO1-HA and DYNAMO1 H116D-HA were expressed at about twofold higher than the endogenous protein (Supplementary Figure 6a and b). Overexpression of DYNAMO1-HA did not affect the division of mitochondria or peroxisomes (Supplementary Figure 6c and d), whereas cells expressing DYNAMO1 H116D-HA showed reductions in division of mitochondria and peroxisomes to an extent similar to that in Dnm1-knockdown cells (Fig. 4a, e; Supplementary Figure 6e-j). Thus, DYNAMO1 H116D apparently functions in a dominant negative fashion. In DYNAMO1 H116D-expressing cells, DYNAMO1 H116D-HA and Dnm1 were properly recruited to the division sites of mitochondria (Fig. 4b; Supplementary Figure 7a and b). However, DYNAMO1 H116D-HA-positive mitochondria were elongated asymmetrically by 1.5–2.0-fold longer than normal mitochondria and exhibited completely impaired constriction, hence appearing as hyper-constricted mitochondria (Fig. 4b–d; Supplementary Figure 7c and d). Similarly, DYNAMO1 H116D-HA-positive peroxisomes had a hyper-constricted phenotype, which were 2–3-fold longer than normal peroxisomes and exhibited stalled constriction (Fig. 4f–h; Supplementary Figure 7e–h). Thus, the H116D mutation impairs complete fission of these two types of organelles. Moreover, in cells expressing *antisense-DYNAMO1*, mitochondria were not constricted and Dnm1 assembly at the division site was significantly reduced (Supplementary Figure 7i–l). Collectively, these data suggest that the GTP-generating activity of DYNAMO1 is essential for complete constriction at organelle division sites, and that adequate amounts of DYNAMO1 appear to be required for Dnm1 recruitment. DYNAMO1 localized to the division sites of both organelles and the entire cytoplasm, suggesting two possibilities of the GTP-providing mechanism. One is local GTP generation at the Dnm1-positive organelle division site and the other is direct regulation of mitochondrial and peroxisomal divisions using a GTP pool in the cytosol. Although DYNAMO1 was localized at organelle division sites and in the entire cytoplasm (Fig. 2), protein translation is likely normal under the expression of DYNAMO1 H116D (Supplementary Figure 8a and b). Thus, inhibition of organelle divisions is supposed to be caused by local GTP generation at organelle division sites rather than depletion of the cytosolic GTP pool. However, DYNAMO1 H116D expression impaired cell cycle progression (Supplementary Figure 8d). Similarly, inhibition of mitochondrial and peroxisomal divisions by supressing Dnm1 also impaired cell cycle progression (Supplementary Figure 8c–e). Therefore, even at a normal cytosolic GTP level, cell cycle progression might be stalled when there are abnormal divisions of mitochondria and peroxisomes, similar to prophase arrest induced by inhibition of plastid division^34^. Interestingly, plastid division occurred under inhibition of DYNAMO1 (Supplementary Figure 8f), although it is also mediated by GTPase dynamin-like protein Dnm2, a homolog of Dnm1^35^. Thus, DYNAMO1 is specifically involved in the divisions of both mitochondria and peroxisomes. In *C. merolae*, at least two genes encode nucleoside diphosphate kinases including DYNAMO1 and its homolog, CMK060c^14^. CMK060c possibly accounts for homeostasis of GTP generation, such as sustaining the global GTP level or compensating for DYNAMO1 functions in the cytosol.

### DYNAMO1 facilitates constriction of Dnm1-based ring

**Fig. 5:**
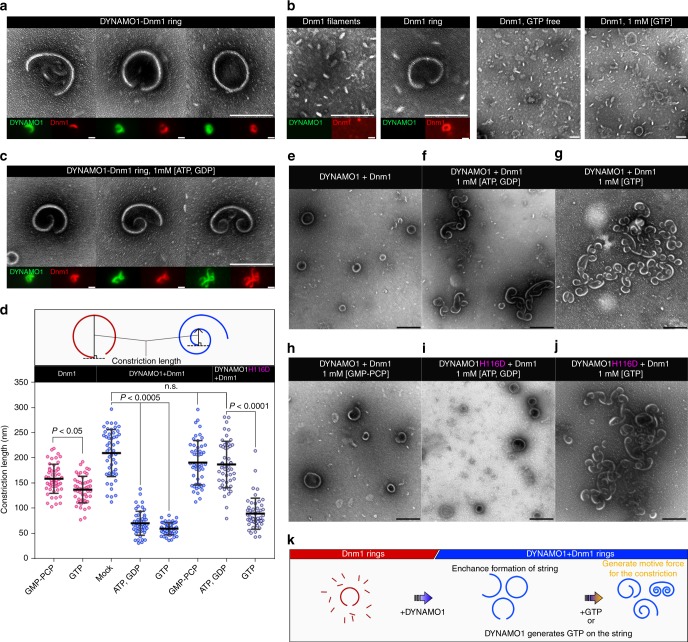
Structure of DYNAMO1-Dnm1 string. **a** Typical structure of DYNAMO1-Dnm1 strings under GTP-free conditions. Immunofluorescence images of DYNAMO1 (green) and Dnm1 (red) are shown in the respective lower panels. **b** Typical structure of DYNAMO1-free Dnm1 filaments and strings under GTP-free condition (left, three panels) or in the presence of GTP (right panel). Immunofluorescence images are shown in the lower panels. **c** Typical structure of DYNAMO1-Dnm1 strings with addition of ATP and GDP. Immunofluorescence images are shown in the lower panels. **d** Schematic image representing measurement of constriction lengths of the strings. Constriction lengths of the DYNAMO1-free Dnm1, DYNAMO1-Dnm1, and DYNAMO1 H116D-Dnm1 strings under the various conditions (*n* = 50). **e**–**j** Structural dynamics of DYNAMO1-Dnm1 or DYNAMO1 H116D-Dnm1 strings upon addition of ATP and GDP, GTP, or GMP-PCP. **k** Schematic image representing the dynamics of DYNAMO1-Dnm1 strings. Scale bars: 200 nm (**a**–**c**); 500 nm (**e**–**j**). Data are means ± s.d. n.s. not significant. *P;* *p*-value (unpaired *t*-test for Dnm1 and DYNAMO1 H116D + Dnm1, Kruskal–Wallis nonparametric ANOVA for DYNAMO1 + Dnm1)

To further investigate mechanisms underlying DYNAMO1 functions in the constriction of mitochondria and peroxisomes, we established an in vitro constriction assay using whole-mount negative-staining electron microscopy. Upon incubation of recombinant DYNAMO1 with Dnm1 under various conditions, single strings (DYNAMO1-Dnm1 strings) were formed with a semi-ring structure and diameter of ~200 nm (Fig. 5a). The strings contained both DYNAMO1 and Dnm1 as assessed by immunofluorescence microscopy (Fig. 5a, lower panels; Supplementary Figure 9a). Substitution of DYNAMO1-free Dnm1 led to significantly less string formation and short filament-like structures with diameter of ~10–20 nm were observed (Fig. 5b; Supplementary Figure 9b). String formation was maximized when the amount of DYNAMO1 molecules was half that of Dnm1 (Supplementary Figure 9c). This result supported the observation that downregulation of DYNAMO1 decreased Dnm1 assembly at mitochondrial division sites (Supplementary Figure 7i–l). Dnm1 of *C. merolae* forms a tetramer with a diameter of ~10 nm in off-membrane state in vitro^36^, and Dnm1 molecules in the division machineries are most likely recruited from membrane-free Dnm1 pool within cytosol^19,25^. Thus, the presence of DYNAMO1 likely induces conformational changes to organize Dnm1 molecules into a highly ordered ring-like structure or stabilizes a ring-like structure at the division site. Because DYNAMO1 showed a GTP-generating activity when supplied with both ATP and GDP (Fig. 1e), we verified whether this enzymatic activity was required for the formation of DYNAMO1-Dnm1 strings. Upon addition of ATP and GDP, the morphology of DYNAMO1-Dnm1 strings was dramatically altered to a spiral shape with one or both tips constricted toward the inside of the string (Fig. 5c–f). The same result was observed with addition of GTP (Fig. 5d, g). In contrast, addition of GMP-PCP did not induce the constriction activity (Fig. 5d, h). Thus, constriction of DYNAMO1-Dnm1 strings depended on the GTPase activity of Dnm1. DYNAMO1-free Dnm1 strings showed little evidence of this structural alteration (Fig. 5d). Collectively, these data indicate that DYNAMO1 is involved in facilitating constriction of the string. When the DYNAMO1-Dnm1 string was replaced by a DYNAMO1 H116D-Dnm1 string (Supplementary Figure 9a), there was no constriction upon addition of ATP or GDP (Fig. 5d, i). However, the DYNAMO1 H116D-Dnm1 string was constricted upon addition of GTP (Fig. 5d, j). Thus, the DYNAMO1 function in constriction of DYNAMO1-Dnm1 strings was independent of the GTP-generating activity. Because the GTPase activity of Dnm1 was higher in DYNAMO1-Dnm1 and DYNAMO1 H116D-Dnm1 strings, in which DYNAMO1 contained half the amount of Dnm1, compared with DYNAMO1-free Dnm1 strings (Supplementary Figure 10), efficient GTP hydrolysis may have generated strong constrictive forces in the DYNAMO1-Dnm1 strings. GTPase activities of Dnm1 and dynamin are increased in an assembly-dependent manner^8,9^. Therefore, DYNAMO1-mediated stabilization of Dnm1 strings likely facilitates GTPase activity of the rings. Taken together, whole-mount morphological observations demonstrated three different DYNAMO1 functions (Fig. 5k): (1) DYNAMO1 enhances formation of the highly ordered structure of Dnm1; (2) DYNAMO1 generates GTP from ATP and GDP adjacent to Dnm1 molecules; (3) DYNAMO1 constructs DYNAMO1-Dnm1 strings that will then be constricted upon hydrolysis of GTP by Dnm1.

## Discussion

**Fig. 6:**
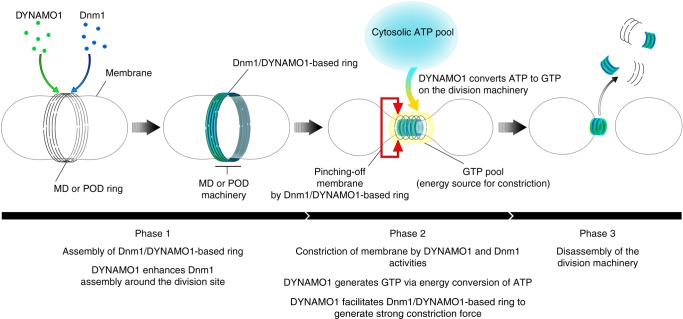
A working model of DYNAMO1 functions during the division of mitochondrion and peroxisomes. Initially, DYNAMO1 is recruited to the division site of the mitochondrion, together with Dnm1 (Phase 1, prophase). DYNAMO1 is involved in Dnm1 assembly around the division site. Next, DYNAMO1 converts ATP to GTP on the ring of the MD machinery. A cytosolic ATP source is used for this reaction. Upon GTP generation, the MD machinery is constricted and GTPase Dnm1 pinches off mitochondrion. Moreover, the DYNAMO1-Dnm1 structure effectively generates a strong motive force for the constriction (Phase 2). MD machinery containing DYNAMO1 and Dnm1 is immediately disassembled (Phase 3). After mitochondrial division, DYNAMO1 functions in the POD machinery during meta-anaphase, in an analogous manner to that in mitochondrial division

Here, we demonstrated that a novel component, DYNAMO1, of MD and POD machineries is an essential molecule for the division of both mitochondria and peroxisomes. We propose three functions of DYNAMO1 in driving these organelle divisions in our working model (Fig. 6). First, the DYNAMO1 molecule is a constituent of MD and POD machineries. DYNAMO1 forms a complex structure with Dnm1 and is involved in Dnm1 assembly at the division site (Phase 1). Second, DYNAMO1 generates GTP on the rings of MD and POD machineries, serving as the energy source most likely provided by consumption of cytosolic ATP for constriction of organelle division machineries (Phase 2). Finally, DYNAMO1 drives constriction of MD and POD machineries (Phase 3). The NDPK domain in DYNAMO1 is conserved among eukaryotes. In flies, dysfunction of nucleoside diphosphate kinase (NDPK/NME) identified in the *Awd* mutant enhances paralysis of the *Shibire* mutant, a temperature-sensitive mutant of dynamin^37^. In mammalian cells, an NDPK ortholog, NME1/NME2, physically interacts with dynamin at the clathrin-coated endocytic pit and facilitates receptor-mediated endocytosis^27,28^. Moreover, NME4 supports dynamin-related GTPase OPA1 that mediates inner mitochondrial membrane fusion^29,30^. Here, we showed that the NDPK ortholog DYNAMO1 is a component of the Dnm1-based organelle division machinery of mitochondria and peroxisomes, which most likely supports local GTP generation to induce the constrictive force. Thus, local GTP generation by the dynamin family member during membrane remodeling is a conserved phenomenon among eukaryotes. Although dynamin family members require considerable amounts of GTP because of the high rate of GTP hydrolysis and lower affinity for GTP^7–9^, cellular free-GTP levels are low (150–300 μM)^38,39^. The local enrichment of GTP is potentially critical to provide a sufficient energy source to drive the supramolecular nanomachineries that influence the dynamic behavior of organelles and cellular structures.

## Methods

### Isolation of MD/PD and POD machineries

For synchronization, *C. merolae* 10D cells were grown in 2 × Allen’s medium. First, cells were subcultured at 1 × 10^7^ cells/mL in 500 mL flat bottles and subjected to a 12-h light/12-h dark cycle (100 μEm^2^/s) at 42 °C under aeration with ordinary air^40^. To isolate MD machinery, synchronized cells were harvested at 13 h after the one set of synchronization. To isolate POD machinery, synchronized cells were cultured under additional treatment with a 1/2500 volume of 100 mM oryzalin stock solution dissolved in dimethyl sulfoxide at 8 h after the one set of synchronization to increase the yield of dividing peroxisomes, then cells were collected at 20 h the one set of synchronization^17^. Harvested cells were centrifuged at 750×*g* for 5 min and resuspended in isolation medium A (20 mM Tris·HCl, pH 7.6, 5 mM MgCl_2_, 30 mM KCl, 5 mM EGTA, 1 mM dithiothreitol (DTT), 3 mg/mL cOmplete EDTA-free protease inhibitor mixture (hereafter cOmplete) (Roche), and 0.18 M sucrose). Resuspended cells were incubated for 30 min at 42 °C in the dark condition. For subcellular fractionation, cells were homogenized using a French Pressure Cell (SLM Aminco) at 600 psi. The homogenate was added with 100 μg/mL DNase I and incubated for 1 h on ice. For the isolation of POD machinery, 0.5% wt/vol Triton X-100 was added. The homogenate (20 mL) were loaded on a Percoll gradient (4 mL of 80% Percoll, 8 mL of 60% Percoll, 6 mL of 40% Percoll, and 2 mL of 0% Percoll). Percoll was dissolved in isolation medium B (20 mM Tris·HCl, pH 7.6, 5 mM MgCl_2_, 30 mM KCl, 5 mM EGTA, 1 mM DTT, 3 mg/mL cOmplete, and 0.3 M sucrose). After centrifugation at 23,000×*g* for 60 min, 2 mL of the organelle fraction at the top of the 40–60% Percoll surface was collected as organelle fraction and then the upper 10 mL was collected as the cytosolic fraction, hence concentration ratio between organelle fraction and cytosolic fraction is about 5 to 1. Organelle fraction was washed twice with isolation medium B without sucrose and incubated on ice for 30 min in 15 mL of isolation medium C (20 mM Tris·HCl, pH 7.6, 5 mM MgCl_2_, 30 mM KCl, 5 mM EGTA, 1 mM DTT, 3 mg/mL cOmplete, 1% Nonidet-P40) on ice for 30 min. The fraction was layered on a 15 mL of 40 % (vol/vol) Percoll and centrifuged at 18,000×*g* for 30 min. To isolate MD/PD machineries, 1.5 mL of the Percoll surface was collected and concentrated to 200 μL in isolation medium C by spinning down. To isolate POD machineries, 1.5 mL of the Percoll surface was mixed vigorously using by vortex and mixed with Opti-prep at final concentration of 42.3%. Then, fraction was centrifuged at 200,000 rpm for 3 h in a NVT65.2 rotor (Beckman Instruments). Fraction 1.0 mL–3.0 mL from the bottom was collected after the centrifugation and scaled down into 200 μL in isolation medium C by spinning down. Collected MD/PD or POD fractions were then mixed with detergents, LDAO (Sigma-Aldrich) and SDS, at final concentration 0.2% and 0.05%, respectively. For observation of machineries, this step was performed after the immunostaining on slide glass at room temperature for 1 min. Protein expression levels of DYNAMO1 in membrane and cytosol fractions were quantified by SDS-PAGE and western blotting using ImageJ. Intensities were calculated by subtracting the background intensity of each lane from the DYNAMO1 intensity. The intensities of DYNAMO1 were normalized to whole cell lysate based on how much concentrated during the fractionation described above.

### Sequences for multiple alignment

The sequences used for multiple alignment were collected by BLAST searches in the databases of the respective species, National Centre for Biotechnology Information, the US Department of Energy Joint Genome Institute (http://genome.jgi-psf.org/), using DYNAMO1 (CML110) of the red alga *C. merolae* as the query. DYNAMO1 (*C. merolae*, CMC110C); DYNAMO1 isoform (*C. merolae*, CMK060C); *Thalassiosira pseudonana*, XP_002295246.1; *Dictyostelium discoideum*, XP_644519.1; *Schisosaccharomyces pombe*, NP_592857.1; *Saccharomyces cerevisiae*, NP_012856.1; *Arabidopsis thaliana*, NP_567346.2; *Caenorhabditis elegans*, NP_492761.1; *Drosophila melanogaster*, NP_476761.3; *Mus musculus*, NP_032730.1, and *Homo sapiens*, NP_937818.1 were aligned.

### Antibodies used for this study

To generate anti-DYNAMO1 antisera in rabbits, mice and rat, the open reading frame of CML110C protein from *C. merolae* was amplified by PCR using the following primers: 5′-cgcGGAyTCCatggaggagacgactttcatcatg-3′ and 5′-cgcCTCGAGagaaacgttctcgtagacccagc-3′ (*Bam*HI and *Xho*I sites are capitalized, respectively). The amplified DNA fragment was cloned into the *Bam*HI and *Xho*I sites of pGEX6p-1 after restriction digestion. The resulting recombinant protein was purified on a GSTrap HP column (GE Healthcare) and subcutaneously injected into rabbits, mice, or rats for immunization (T.K. Craft Corp.). DYNAMO1 antibodies are used for immunofluorescence microscopy (1/3000 dilution) and for immunoblotting (1/2000 dilution). The other antibodies used in this study were a rabbit anti-Dnm1 antibody^19^ (1/2000 dilution for immunofluorescence microscopy and immunoblotting), mouse Mda1 antibody^41^ (1/400 for immunofluorescence microscopy and 1/1000 for immunoblotting), rat EF-Tu antibody^42^ (1/1000 for immunofluorescence microscopy and immunoblotting), mouse FtsZ1 antibody^20^ (1/100 for immunofluorescence microscopy), guinea pig anti-EF1α antibody^43^ (1/2000 for immunoblotting), guinea pig anti-porin antibody^44^ (1/400 for immunoblotting), rat anti-PDR1 antibody^21^ (1/200 for immunofluorescence microscopy), and rabbit Pex14 antibody^25^ (1/1000 for immunoblotting). The Alexa 488-conjugated mouse anti-HA antibody was purchased from Thermo Fisher Scientific (Cat. #A-21287). A mouse anti-GFP antibody (B2) was purchased from Santa Cruz Biotechnology (Cat. #sc-9996). For immunoblotting, the secondary antibody was alkaline phosphatase-conjugated rabbit and mouse IgG (BioRad). Original data of immunoblotting with these antibodies are shown in Supplementary Figure 11. For immunofluorescence microscopy, the secondary antibodies were Alexa Fluor 350- (mouse; Cat. #A-11045, rabbit; Cat. #A-21068, rat; Cat. #A-21093) (1/200 dilution), Alexa Fluor 488-(mouse; Cat. #A-11001, rabbit; Cat. #A-11008, rat; Cat. #A-11006) (1/1,000 dilution) and Alexa Fluor 555-(mouse; Cat. #A-21322, rabbit; Cat. #A-21428, rat; Cat. #A-21434) (1/1,000 dilution) conjugated IgGs (Thermo Fisher Scientific). For immunoelectron microscopy, mouse and rabbit 10-nm (Cat. #810.177) (1/40 dilution) or 15-nm (Cat. #815.166) (1/20 dilution) immunogold particle-conjugated IgGs were purchased from Aurion.

### Plasmid construction

To construct a plasmid for expression of recombinant Dnm1 protein, the Dnm1 (CME019) sequence was amplified from *C. merolae* genomic DNA with the following primers: 5′-cgcGGATCCatggagcgcctaatacctatcg-3′ (*Bam*HI site is capitalized) and 5′-cgcGAATTC aatctcctctttgacgtgaacg-3′ (*Eco*RI site is capitalized). The amplified fragment was digested with *Bam*HI and *Eco*RI (Nippon Gene) and inserted into pGEX6p-1. To prepare a plasmid for transient gene expression, the *DYNAMO1* (*CML110*) sequence, including the upstream ~500 bp sequence, was amplified with the following primers: 5′-ctctcgaggcagaggaatgtggacagtcag-3′ and 5′-ttgctcacagaaacgttctcgtagacccagc-3′. The amplified fragments were combined with pTH-2PL^45^ linearized with primers 5′-acgtttctgtgagcaagggcgaggag-3′ and 5′-cctctgcctcgagagcttggcactgg-3′ using an In-Fusion HD cloning kit (Clontech). A triple tandem HA-tagged sequence (3 × HA-tag) was inserted into the C-terminus of DYNAMO1 by PCR with the following primers: 5′-TATGACGTCCCGGACTATGCAGGATACCCTTATGACGTTCCAGATTACGCTtaaatcgttcaaacatttggcaataaag-3′ and 5′-AGTCCGGGACGTCATAGGGATAGCCCGCATAGTCAGGAACATCGTATGGGTAagaaacgttctcgtagacccagc-3′ (3 × HA-tag is capitalized). The product was circularized using the In-Fusion HD cloning kit. The antisense-DYNAMO1 sequence was amplified from *C. merolae* genomic DNA with the following primers: 5′-cgcGGATCCgtcattgacgcctcagatagcgtggagag-3′ (*Bam*HI site is capitalized) and 5′-cgcAAGCTTatggaggagacgactttcatcatg-3′ (*Hin*dIII site is capitalized). The amplified fragment was treated with *Bam*HI and *Hin*dIII and inserted into pMito-GFP^17^. The antisense Dnm1 plasmid co-expressing mCherry is described elsewhere^25^. To generate DYNAMO1 with the H116D mutation, a plasmid encoding DYNAMO1 was amplified with the following primers: 5′-gtcattgacgcctcagatagcgtggagag-3′ and 5′-tgaggcgtcaatgacgtttcgccctacg-3′. The amplified linear construct harboring His-116 replaced by Asp was recovered using the In-Fusion HD cloning kit, as described for the DYNAMO1 expressing plasmid.

### Purification of DYNAMO1

For DYNAMO1 expression, the plasmid encoding GST-tagged DYNAMO1 used for antibody generation was employed as described above. For DYNAMO1 H116D expression, the GST-tagged DYNAMO1 plasmid was amplified with the same primers to generate the plasmid for transient expression of the H116D mutant described above, and the amplified fragment was cloned by infusion cloning. XL-1 Blue strain cells were transformed with these plasmids, cultured at 37 °C for 12 h in 100 mL Luria Bertani (LB) medium, scaled up to 1 L LB medium, and incubated further at 37 °C for 2 h and then at 18 °C for 1 h. Isopropyl β-d-1 thiogalactopyranoside (IPTG) was added at a final concentration of 0.1 mM, and cells were harvested after a further 12 h of incubation at 18 °C by centrifugation at 1000×*g* for 10 min. Cell pellets were resuspended in 200 mL HEPES buffer (HB250) containing 250 mM NaCl, 20 mM HEPES-KOH, pH 7.5, 2 mM EGTA, 1 mM MgCl_2_, 1 mM DTT, and a cOmplete. After homogenization by sonication for 10 min, the supernatant was filtered and rotated at 4 °C for 1 h with 1 mL glutathione-Sepharose 4B beads (GE Healthcare). The beads were collected, washed, and resuspended in 10 mL HB250. Then, the beads were loaded onto a 10 mL Poly-Prep Chromatography column (BioRad) and washed with HBs: 5 mL HB250, 10 mL HB100 containing 100 mM NaCl, and 20 mL cOmplete-free HB50 containing 50 mM NaCl. To remove GST-tag, Sepharose beads were treated with PreScission Protease (GE Healthcare) at 4 °C for 2 days, and DYNAMO1 was eluted.

### Purification of Dnm1

For Dnm1 expression, pGex6p-1Dnm1^25^ was introduced into the *Escherichia coli* XL-1 Blue strain. Transformed XL-1 Blue strain cells were cultured at 37 °C for 12 h in 100 mL of LB medium, scaled up to 1 L of LB medium, and further incubated at 37 °C for 2 h and at 18 °C for 1 h. IPTG was added to a final concentration of 0.1 mM, and cells were harvested after a further 8-h incubation at 18 °C by centrifugation at 1000×*g* for 10 min. Cell pellets were resuspended in 100 mL of HB150. After homogenization by sonication, the supernatant was filtered and rotated at 4 °C with 1 mL of glutathione Sepharose 4B beads (GE Healthcare) for 1 h. The beads were collected and resuspended in 10 mL of HB100. Then, beads were loaded onto a 10 mL Poly-Prep Chromatography column (BioRad) and washed with cOmplete-free HB75 and 20 mL of complete-free HDB50. After washing with HBs, Sepharose beads were treated with PreScission Protease (GE Healthcare) at 4 °C for 2 days and the cleaved-off proteins were eluted.

### Gel filtration assay of recombinant DYNAMO1

A total of 1 μg purified recombinant DYNAMO1 was analyzed by gel filtration on a Sepharose 6 10/300 GL column using an AKTA prime system (GE Healthcare). Gel filtration standards (BioRad Laboratories) including thyroglobulin (669 kDa), γ-globulin (158 kDa), and ovalbumin (45 kDa) were used to calculate the molecular mass of DYNAMO1. Partition coefficient *K*_av_ was calculated by

$$\frac{{(V_{{\mathrm{e}}({\mathrm{B}})} - V_0)}}{{V_{\mathrm{c}} - V_0}} = K_{{\mathrm{av}}({\mathrm{B}})}$$

where *V*_e(B)_ is the elution volume, *V*_0_ is the void volume, and *V*_c_ is the total geometric volume of the column, 24 mL.

### Pull-down assay and immunoprecipitation

Synchronized cells at M phase (16 h after the one set of light/dark cycle) were lysed in binding buffer (50 mM HEPES-KOH, pH 7.5, 0.15 M NaCl, 0.5% Triton X-100, 1 mM EDTA, 1 mM DTT, a cOmplete). Cell lysates were centrifuged at 20,000×*g* for 5 min at 4 °C to remove insoluble cell debris and resulting supernatants were pre-incubated with glutathione-Sepharose beads (GE Healthcare) or protein A Sepharose CL-4B (GE Healthcare) for 1 h at 4 °C to reduce nonspecific binding to the Sepharose beads. For pull-down assay, the supernatants or recombinant Dnm1 were incubated with purified GST-DYNAMO1 for 3 h at 4 °C. The protein complexes were recovered by incubating for 1 h at 4 °C with glutathione-Sepharose beads and eluted with Laemmli sample buffer. For immunoprecipitation, the supernatants were incubated with anti-Dnm1 antibody at 4 °C for overnight. Antibody-antigen complexes were recovered by incubating for 1 h at 4 °C with Protein A-Sepharose CL-4B and eluted with Laemmli sample buffer.

### Phase contrast and immunofluorescence microscopies

*C. merolae* cells were fixed at −30 °C for 5 min with 1% wt/vol paraformaldehyde and 10% DMSO dissolved in 100% methanol^42^. Phase contrast and immunofluorescence images were captured with a fluorescence microscope (BX51; Olympus). Immunofluorescence profiles were acquired with ImageJ software (National Institutes of Health, Bethesda, MD, USA).

### LC–MS/MS analysis of isolated POD machinery

Proteins in isolated POD machinery and interphase fractions were separated by SDS-PAGE and stained with silver stain (0.02% Na_2_S_2_O_3_, 0.1% AgNO_3_, and 0.04% formalin/2% Na_2_CO_3_; Sigma-Aldrich). Signal intensities of silver-stained bands were compared using the ImageJ plot profile between POD machinery and interphase fractions. Distinct bands with signal intensity ratios higher than 1 of POD machinery to interphase fractions were cut out, reduced with DTT, alkylated with iodoacetamide, digested with trypsin, and analyzed by an ion-trap mass spectrometer equipped with a nano-LC electrospray ionization source at the Human Proteome Research Centre, Kyushu University, Japan^46^. The obtained peptides were dried and then dissolved in a solution containing 0.1% trifluoroacetic acid and 2% acetonitrile before nanoscale liquid chromatography (nanoLC)–MS/MS analysis with a system consisting of an LTQ Orbitrap Velos Pro mass spectrometer (Thermo Fisher Scientific) coupled with a nanoLC instrument (Advance LC, Michrom BioResources) and HTC-PAL autosampler (CTC Analytics). Peptide separation was performed with an in-house pulled and fused silica capillary (internal diameter, 0.1 mm; length, 10 cm; tip internal diameter, 0.05 mm) packed with a C18 L-column material (average particle size, 3 μm) (Chemicals Evaluation and Research Institute). The mobile phases were 0.1% formic acid in water (A) and 100% acetonitrile (B). Peptides were eluted with linear gradients of 5–40% B for 20 min and 40–95% B for 1 min, and then kept at 95% B for 4 min at a flow rate of 300 nL/min. Collision-induced dissociation (CID) spectra were acquired automatically in a data-dependent scan mode with the dynamic exclusion option. Full MS spectra were obtained with Orbitrap in the mass/charge (*m*/*z*) range of 300–2000 with a resolution of 60,000 at *m*/*z* 400. The nine most intense precursor ions (minimum ion count threshold of 1000) in the full MS spectra were selected for subsequent ion-trap MS/MS analysis with the automated gain control (AGC) mode. The AGC was set to 1 × 10^6^ for full MS and 1 × 10^4^ for CID MS/MS. The normalized collision energy value was set to 35%. The lock mass function was activated to minimize mass error during analysis. Peak lists were generated by MSn.exe (Thermo Fisher Scientific) with a minimum scan/group value of 1 and compared with the in-house-curated target/decoy *C. merolae* genome database (http://merolae.biol.s.u-tokyo.ac.jp/) using the MASCOT algorithm (ver. 2.4.1). Trypsin was selected as the enzyme, the allowed number of missed cleavages was set at 1, and carbamidomethylation on cysteine was selected as the fixed modification. Oxidized methionine was searched as the variable modification. Precursor mass tolerance was 10 ppm and the tolerance of MS/MS ions was 0.8 Da. The threshold used for peptide identification was a MASCOT score of ≥30 and a delta score (the difference between first- and second-assigned peptides) of ≥ 18. Using these criteria, the false positive rate was <1%. Contaminants of plastid genome-encoded abundant photosynthetic proteins with a MASCOT score of ≥100 were excluded from the data.

### LC–ESI–MS/MS analysis of nucleotides

To measure DYNAMO1 activity for nucleotide exchange, 100 nM recombinant DYNAMO1 described above was mixed with nucleotides and incubated at 37 °C for 3 min. The reaction mixture was immediately mixed with 1.25 × and 2.5 × volumes of chloroform and methanol, respectively. Subsequently, 2 mM raffinose, as an internal standard used for normalization between samples, was added, and all samples were lyophilized and dissolved in methanol:water (1:1 v/v). After centrifugation at 10,000×*g* for 5 min, the supernatant was evaporated at room temperature under nitrogen for 6 h and then subjected to LC–ESI–MS/MS analysis. LC–ESI–MS/MS was performed using a 4000 QTRAP quadrupole linear ion-trap hybrid mass spectrometer (AB Sciex) with an ACQUITY UPLC System (Waters). Samples were injected into an ACQUITY UPLC BEH C18 column (1 × 150 mm; Waters) and then directly subjected to ESI-MS/MS analysis. A 10 μL aliquot was separated by step gradient elution with mobile phases A (water, 0.1% formic acid and 0.028% ammonia) and B (20% acetonitrile, 0.1% formic acid, and 0.028% ammonia) at the following ratios: 95:5 (for 0–5 min), 0:100 (5–25 min), 0:100 (25–30 min), and 95:5 (30–40 min). The flow rate was 50 μL/min at 30 °C. The source temperature was 400 °C, the declustering potential was −85, and the collision cell exit potential was −11. The ion transitions at *m*/*z* 506 > 79, 426 > 79, 522 > 79, 442 > 79, and 503 > 179 for ATP, ADP, GTP, GDP, and raffinose, respectively, were used in the multiple reaction monitoring mode. Data were analyzed and quantified using Analyst software (AB Sciex). Nucleotides were purchased from Sigma-Aldrich.

### Electron microscopy

Whole-mount immunoelectron microscopy observations of POD machinery, DYNAMO1 and Dnm1 structures was performed by transmission election microscopy (Hitachi). The purified proteins were mixed and incubated at 4 °C overnight and then incubated with various nucleotides at 37 °C for 5 min. The mixtures were mounted on formvar coated 200-mesh copper grids (Nisshin EM Co., Ltd.) that had been carbonized and glow discharged immediately prior to use. The grids were negatively stained with 2% uranyl acetate and observed at 80 kV. Nucleotides and non-hydrolysable analogs were purchased from Sigma-Aldrich.

### Kinetic analysis of DYNAMO1-Dnm1 complexes

Assays of GTPase activities of DYNAMO1 (250 nM) and Dnm1 (500 nM) were performed at 37 °C in reaction mixtures (20 mM HEPES, pH 7.5, 30 mM KCl, 1 mM MgCl_2_ and 1 mM DTT). Released phosphate was detected by Malachite Reagent (BioAssay Systems). The absorbance at 600 nm was measured with a microplate reader (Benchmark Plus).

### Quantification and statistical analysis

Experiments were performed using at least two independent cultures unless noted otherwise. The profiles of organelle divisions and cell cycles were chosen randomly to sample unbiased populations. Division sites are defined as regions with Dnm1 particle-positive sites or morphologically constricted sites. We used unpaired *t*-test for normal distribution data. We used the Mann–Whitney test or Kruskal–Wallis nonparametric ANOVA for skewed distribution of the data. *p*-values in multiple comparison are adjusted with Bonferroni correction. The skewness was determined by Pearson’s skewness test in GraphPad Prism 7.4. Confidence levels are shown in each graph.

## Electronic supplementary material

Reporting Summary

Supplementary Information

## Data Availability

All relevant data are available from the corresponding author upon reasonable request. The full set of acquired data and results from MS analysis are available in jPOSTrepo (Japan ProteOme STandard Repository, https://repository.jpostdb.org/). The accession numbers are PXD010716 for Proteomechange and JPST000474 for jPOST. A reporting summary for this Article is available as a Supplementary Information file.
