# Supplementary material for: Onsite GTP fuelling via DYNAMO1 drives division of mitochondria and peroxisomes
Source: Nat Commun. 2018 Nov 6;9:4634. doi: 10.1038/s41467-018-07009-z (PMC6219506; doi:10.1038/s41467-018-07009-z)
Supplement: Supplementary file 1 — Reporting Summary [file 41467_2018_7009_MOESM1_ESM.pdf]

nature research

Corresponding author(s): Yukio Fujiki

Reporting Summary

Nature Research wishes to improve the reproducibility of the work that we publish. This form provides structure for consistency and transparency in reporting. For further information on Nature Research policies, see Authors & Referees and the Editorial Policy Checklist.

Statistical parameters

When statistical analyses are reported, confirm that the following items are present in the relevant location (e.g. figure legend, table legend, main text, or Methods section).

n/a	Confirmed	
☐	☒	The exact sample size (n) for each experimental group/condition, given as a discrete number and unit of measurement
☐	☒	An indication of whether measurements were taken from distinct samples or whether the same sample was measured repeatedly
☐	☒	The statistical test(s) used AND whether they are one- or two-sided<br>*Only common tests should be described solely by name; describe more complex techniques in the Methods section.*
☒	☐	A description of all covariates tested
☐	☒	A description of any assumptions or corrections, such as tests of normality and adjustment for multiple comparisons
☐	☒	A full description of the statistics including central tendency (e.g. means) or other basic estimates (e.g. regression coefficient) AND variation (e.g. standard deviation) or associated estimates of uncertainty (e.g. confidence intervals)
☐	☒	For null hypothesis testing, the test statistic (e.g. F, t, r) with confidence intervals, effect sizes, degrees of freedom and P value noted<br>*Give P values as exact values whenever suitable.*
☒	☐	For Bayesian analysis, information on the choice of priors and Markov chain Monte Carlo settings
☒	☐	For hierarchical and complex designs, identification of the appropriate level for tests and full reporting of outcomes
☐	☒	Estimates of effect sizes (e.g. Cohen's d, Pearson's r), indicating how they were calculated
☐	☒	Clearly defined error bars<br>*State explicitly what error bars represent (e.g. SD, SE, CI)*

Our web collection on statistics for biologists may be useful.

Software and code

Policy information about availability of computer code

Data collection	LC–ESI–MS/MS for nucleotide analysis was performed by Analyst 1.4.2 (sciex).
Data analysis	Immunofluorescence profiles were acquired with ImageJ software 1.46r (National Institutes of Health, Bethesda, MD, USA)<br>Protein identification and calculation of exponentially modified protein abundance index (emPAI) values were performed using the MASCOT (Matrix Science) running on the local server using the C. merolae genome database (http://merolae.biol.s.u-tokyo.ac.jp/)<br>LC–ESI–MS/MS analysis of nucleotides was performed by Analyst 1.4.2 (sciex).<br>Statical analysis and Pearson's skewness test was performed by GraphPad Prism 7.4.

For manuscripts utilizing custom algorithms or software that are central to the research but not yet described in published literature, software must be made available to editors/reviewers upon request. We strongly encourage code deposition in a community repository (e.g. GitHub). See the Nature Research guidelines for submitting code & software for further information.

Data

Policy information about availability of data

All manuscripts must include a data availability statement. This statement should provide the following information, where applicable:

- Accession codes, unique identifiers, or web links for publicly available datasets
- A list of figures that have associated raw data
- A description of any restrictions on data availability

All data and the data availability statement were described in manuscript.
The full set of acquired data and results from mass spec for protein analysis are available in jPOSTrepo (Japan ProteOme STandard Repository, https://repository.jpostdb.org/).

Field-specific reporting

Please select the best fit for your research. If you are not sure, read the appropriate sections before making your selection.

☒ Life sciences ☐ Behavioural & social sciences ☐ Ecological, evolutionary & environmental sciences

For a reference copy of the document with all sections, see nature.com/authors/policies/ReportingSummary-flat.pdf

Life sciences study design

All studies must disclose on these points even when the disclosure is negative.

Sample size	All sample sizes are listed in detail in the figure legends and main text.
Data exclusions	No data were excluded from the analyses.
Replication	All data presented were from biological replicates. All attempts at replication were successful.
Randomization	Data were collected and chosen randomly to sample unbiased populations.
Blinding	Experimenters were performed under the blinded condition to exclude bias.

Reporting for specific materials, systems and methods

Materials & experimental systems

n/a	Involved in the study	
☐	☒	Unique biological materials
☐	☒	Antibodies
☒	☐	Eukaryotic cell lines
☒	☐	Palaeontology
☒	☐	Animals and other organisms
☒	☐	Human research participants

Methods

n/a	Involved in the study	
☒	☐	ChIP-seq
☒	☐	Flow cytometry
☒	☐	MRI-based neuroimaging

Unique biological materials

Policy information about availability of materials

Obtaining unique materials	C. marolae are readily available from the authors

Antibodies

Antibodies used	Dnm1 rabbit antibody, Mda1 mouse antibody, EF-Tu rat antibody, FtsZ1 mouse antibody, EF1α guinea pig antibody, porin guinea pig antibody, PDR1 rat antibody, Pex14 rabbit antibody, DYNAMO1 rat, rabbit, or mouse antibodies were used.
Validation	All antibodies have been previously validated for use in the system under immunofluorescence and western blotting as shown in

the cited articles. DYNAMO1 antibody is validated in this study.
