# Supplementary material for: Onsite GTP fuelling via DYNAMO1 drives division of mitochondria and peroxisomes
Source: Nat Commun. 2018 Nov 6;9:4634. doi: 10.1038/s41467-018-07009-z (PMC6219506; doi:10.1038/s41467-018-07009-z)
Supplement: Supplementary file 2 — Supplementary Information [file 41467_2018_7009_MOESM2_ESM.pdf]

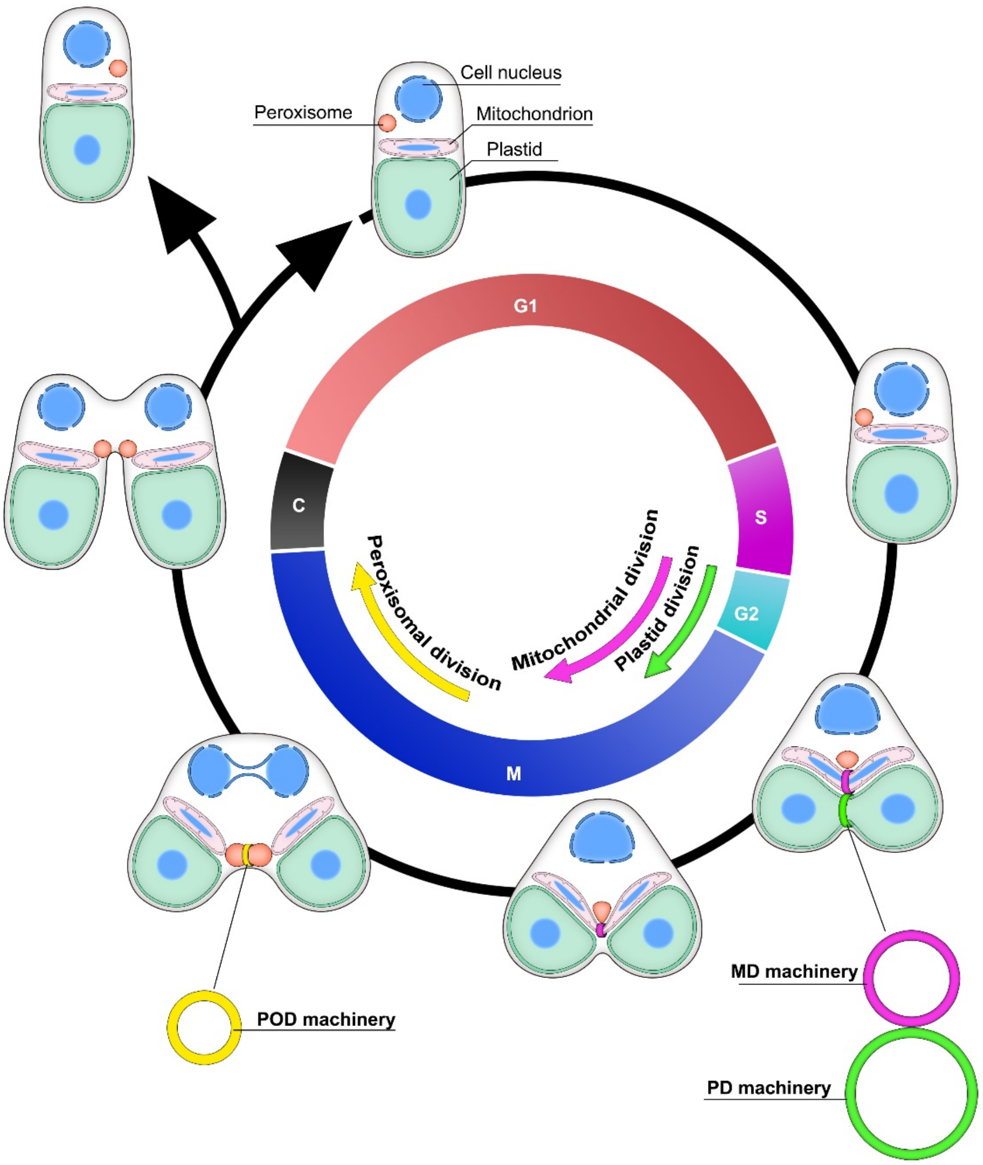

Supplementary Figure 1. Schematic representation of division timing of cell nuclei, mitochondria, peroxisomes and plastids during the cell cycle. The duration of the mitotic cycle of *C. merolae* is ~22 h. Mitochondrial and plastid divisions occur during G2-early mitotic phase (prophase) by contraction of the mitochondrial division (MD) and plastid dividing (PD) machineries. Subsequently, peroxisomal division occurs during late mitotic phase (metaphase–anaphase) by contraction of the peroxisome-dividing (POD) machinery. After mitosis, respective daughter organelles are partitioned to the daughter cells and physically separated by cytokinesis. G1, G1 phase; S, synthesis phase; G2, G2 phase; M, mitotic phase; C, cytokinesis.

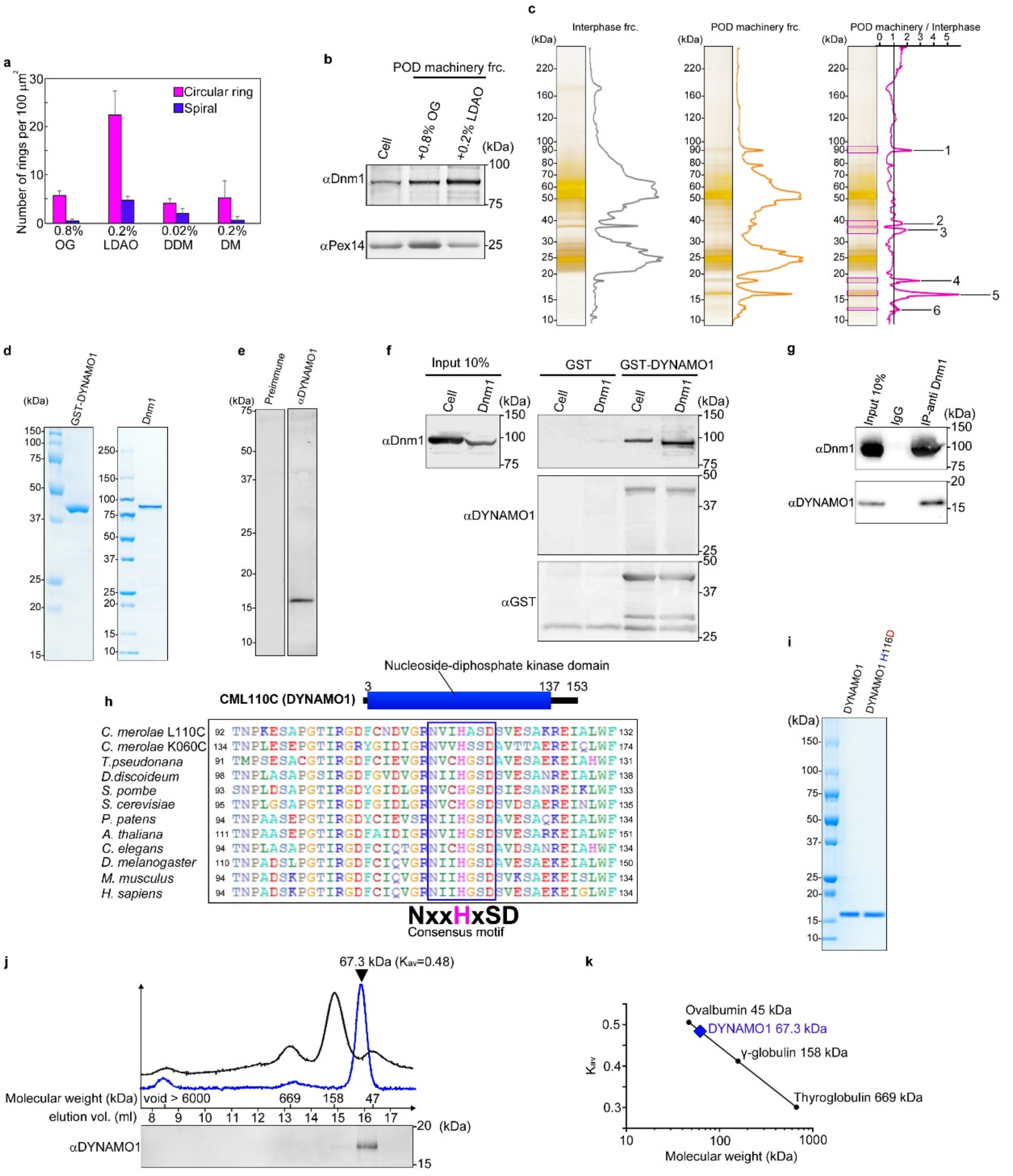

Supplementary Figure 2. Isolation and characterization of DYNAMO1. (**a**) The number of circular and spiral POD machineries in fractions treated with various detergents. OG; n-octyl-β-D-glucoside, LDAO; lauryldimethylamine-N-oxide, DDM; n-dodecyl-β-D-maltoside, DM; Decyl β-D-maltopyranoside. n = 5. (**b**) Immunoblotting analysis of Dnm1 and Pex14 (a peroxisomal membrane protein) in whole cells or in isolated POD machinery fractions treated with 0.8% OG or 0.2% LDAO. (**c**) SDS-PAGE analysis of proteins in the POD machinery isolated from cells during interphase (negative control) and peroxisomal division periods. Enriched bands in POD machinery are characterized by calculating intensity ratio of POD machinery fraction to interphase fraction. Gray and yellow curve lines represent scanning plot profiles of silver staining. Purple line represents

the ratio of plot profiles POD to the control interphase. Purple boxed are six distinct bands enriched in POD machinery fraction and analysed by LC-MS/MS (see Supplementary Table 1). n = 1. (**d**) CBB-stained SDS-PAGE gel images of the recombinant GST-fused DYNAMO1 and Dnm1 used in this study. (**e**) Immunoblotting analysis with preimmune and anti-DYNAMO1 antibodies of whole cell lysates of *C. merolae* during mitosis. (**f**) Pull-down assay of GST–DYNAMO1 with whole cell lysates of *C. merolae* and recombinant Dnm1. (**g**) Immunoprecipitation assay with anti-Dnm1 antibody using whole cell lysates of *C. merolae* during mitosis. (**h**) Predicted domain architecture of CML110C/DYNAMO1 and sequence alignment of its homologous proteins. (**i**) CBB-stained SDS-PAGE gel images of the recombinant DYNAMO1 and DYNAMO1 H116D used in this study. (**j** and **k**) Gel filtration analysis of recombinant DYNAMO1. Eluting profiles of molecular mass markers and recombinant DYNAMO1 are shown in black and blue lines, respectively. Data are means ± s.d.

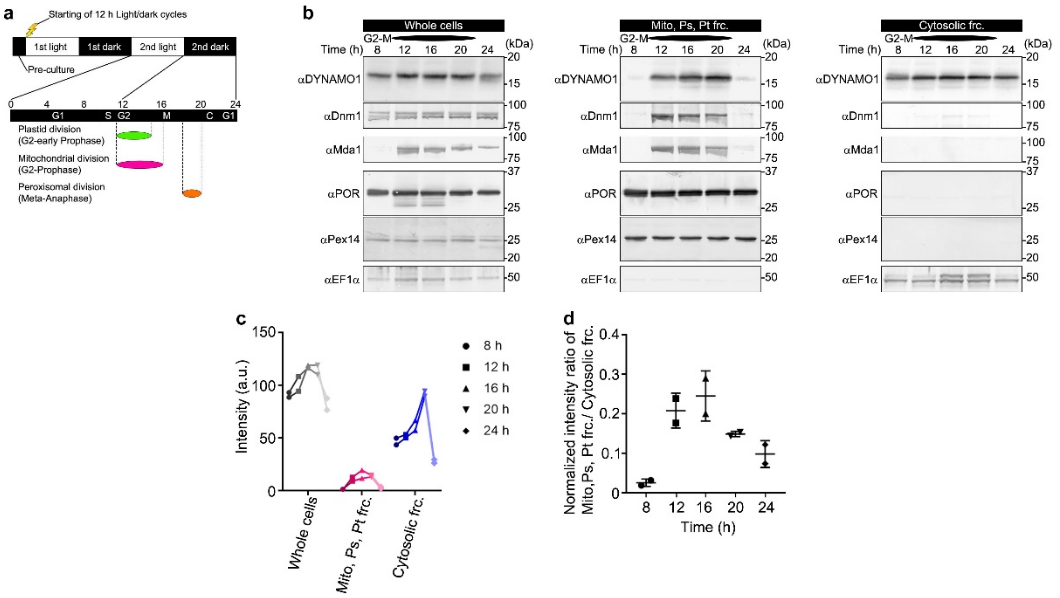

Supplementary Figure 3. Expression levels of DYNAMO1 during the cell cycle. (**a**) Schematic image representing the time course of synchronization. G1, G1 phase; S, synthesis phase; G2, G2 phase; M, mitotic phase; C, cytokinesis. (**b**) Protein expression throughout the cell cycle, as assessed by immunoblotting for DYNAMO1, Dnm1, Mda1, porin (POR), Pex14 and EF-Tu in whole cells, in organelle fractions containing mitochondrion, peroxisome and plastid (Mito, Ps, Pt frc), and in cytosolic fractions. Ratio of concentration between organelle fraction and cytosolic fraction is 5:1. (**c**) Protein expression level of DYNAMO1 in whole cell, organelle fractions and cytosolic fractions throughout the cell cycle. Line graphs of two independent experiments are shown. (**d**) Normalized ratio of DYNAMO1 expression level in organelle fractions versus cytosolic fractions. Two plots at respective time points in two independent two experiments are shown. Means ± s.d., *n*=2.

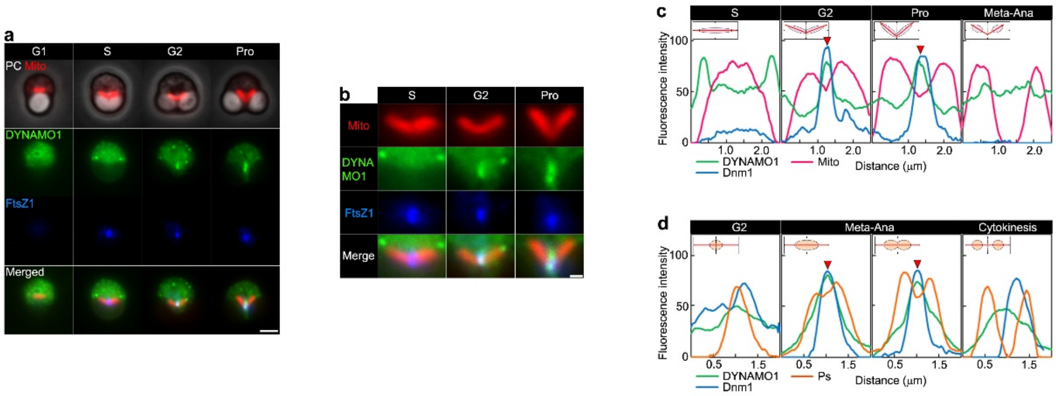

Supplementary Figure 4. Localization of DYNAMO1 during the cell cycle. (**a**) Phase contrast and immunofluorescence images of mitochondrion (Mito), DYNAMO1 and FtsZ in cells during various cell cycle stages. (**b**) Magnified images around a mitochondrion in (a). (**c**) Line-scan analysis of fluorescence intensity verifying DYNAMO1 localization on the mitochondrion in images of Figure 2a. (**d**) Line-scan analysis of fluorescence intensities verifying DYNAMO1 localization on the peroxisome in images of Figure 2e. Scale bars, 1 μm (a); 500 nm (b).

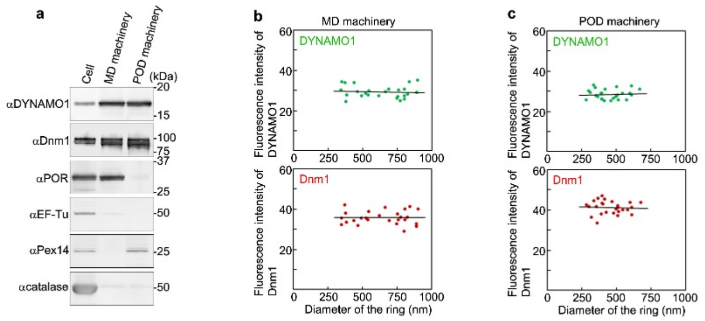

Supplementary Figure 5. Characteristics of DYNAMO1 in MD and POD machineries. (**a**) Immunoblotting analyses of DYNAMO1, Dnm1, POR, EF-Tu, Pex14 and catalase in the whole cells and in fractions of MD and POD machineries. (**b**) Immunofluorescence intensities of DYNAMO1 and Dnm1 on the rings, of various diameters, in MD machineries (n = 25). (**c**) Immunofluorescence intensities of DYNAMO1 and Dnm1 on rings, of various diameters, in POD machineries (n = 25).

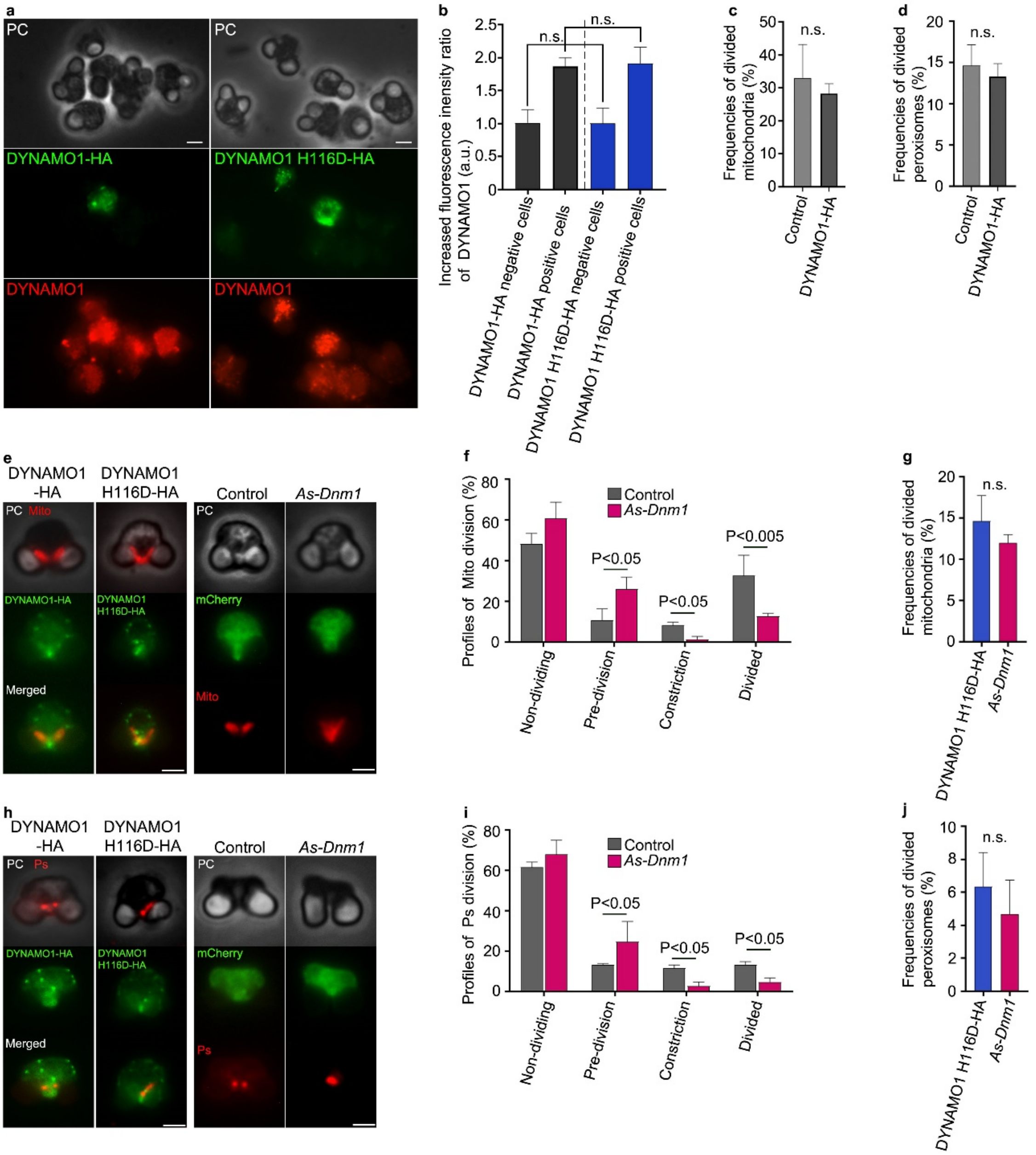

Supplementary Figure 6. Inhibition of division by inactive DYNAMO1 and Dnm1 knockdown. (**a** and **b**) Expression level of DYNAMO1 and DYNAMO1 H116D is compared to the endogenous level by quantifying immunofluorescence intensity of cells stained with anti-DYNAMO1 antibody. Increased fluorescence intensities are measured by subtracting the intensity ratio of DYNAMO1 in non-transfected cells from transfected cells and normalized to non-transfected cells in *DYNAMO1-HA*- or *DYNAMO1 H116D-HA*-transfected cell cultures. (**c**) Frequencies of divided mitochondria at 24 h after the transfection of DYNAMO1-HA or control plasmid. (**d**) Frequencies of divided peroxisomes at 24 h after the transfection of DYNAMO1-HA or control plasmid. (**e**) Phase-contrast and immunofluorescence images of mitochondria under the expression of DYNAMO1 H116D-HA or antisense (*As*)-*Dnm1*. (**f**) Profiles of mitochondrial division at 24 h after

the transfection of *As-Dnm1*. (**g**) Frequencies of divided mitochondria 24 h after the transfection of DYNAMO1 H116D-HA or *As-Dnm1*. (**h**) Phase-contrast and immunofluorescence images of peroxisomes under the expression of DYNAMO1 H116D-HA or *As-Dnm1*. (**i**) Profiles of peroxisomal division at 24 h after the transfection of *As-Dnm1*. (**j**) Frequencies of divided peroxisomes at 24 h after the transfection of DYNAMO1 H116D-HA or *As-Dnm1*. $n = 2$. At least 50 cells are examined in each experiment. Data are means ± s.d. Scale bars, 1 μm. n.s.; not significant, P; p-value (b; unpaired *t*-test, c, d, f, g i, j; Mann-Whitney test).

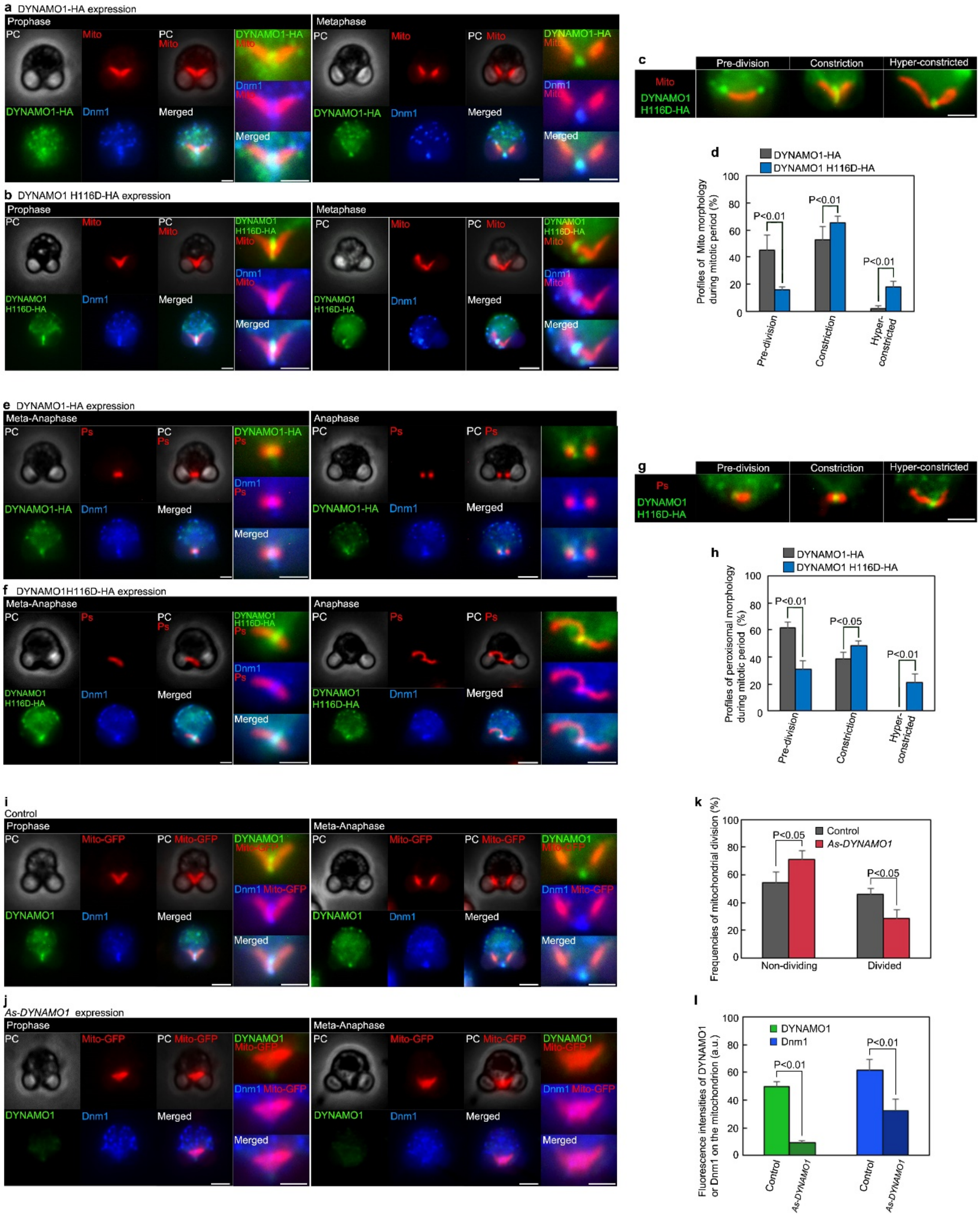

Supplementary Figure 7. Typical localization of DYNAMO1-HA and DYNAMO1 H116D-HA. (**a** and **b**) Phase contrast and immunofluorescence images of mitochondrion, DYNAMO1-HA or DYNAMO1 H116D-HA and Dnm1 in cells expressing DYNAMO1-HA, during prophase and metaphase. (**c**) Typical mitochondrial morphology in cells expressing DYNAMO1 H116D-HA. (**d**) Percentages of mitochondrial morphology in cells expressing DYNAMO1-HA or DYNAMO1 H116D-HA. (**e** and **f**) Phase contrast and immunofluorescence images of peroxisomes, DYNAMO1-HA or DYNAMO1 H116D-HA and Dnm1 in cells expressing DYNAMO1-HA during

meta–anaphase. (**g**) Typical peroxisomal morphology in cells expressing DYNAMO1 H116D. (**h**) Percentages of peroxisomal morphology in cells expressing DYNAMO1-HA or DYNAMO1 H116D-HA. (**i** and **j**) Phase contrast and immunofluorescence images of mitochondrion transit peptide fused GFP (Mito–GFP, anti-GFP antibody), DYNAMO1 and Dnm1 in cells expressing Mito–GFP (control) or antisense-DYNAMO1 plus Mito–GFP (*antisense*-DYNAMO1) during prophase and meta–anaphase. (**k**) Percentages of mitochondrial division in control cells and *Antisense* (*AS*)-DYNAMO1 cells. n = 3. Fifty cells were examined in each experiment. (**l**) Immunofluorescence intensities (a.u., auxiliary unit) of DYNAMO1 and Dnm1 in control and *Antisense* (*AS*)-DYNAMO1 cells. Scale bars, 1 μm; 500 nm (magnified images shown on the right end of the respective panels). n = 3. Twenty-five cells were examined in each experiment. Data are means ± s.d. P; p-value (d, h, k; Mann-Whitney test, i; unpaired t-test).

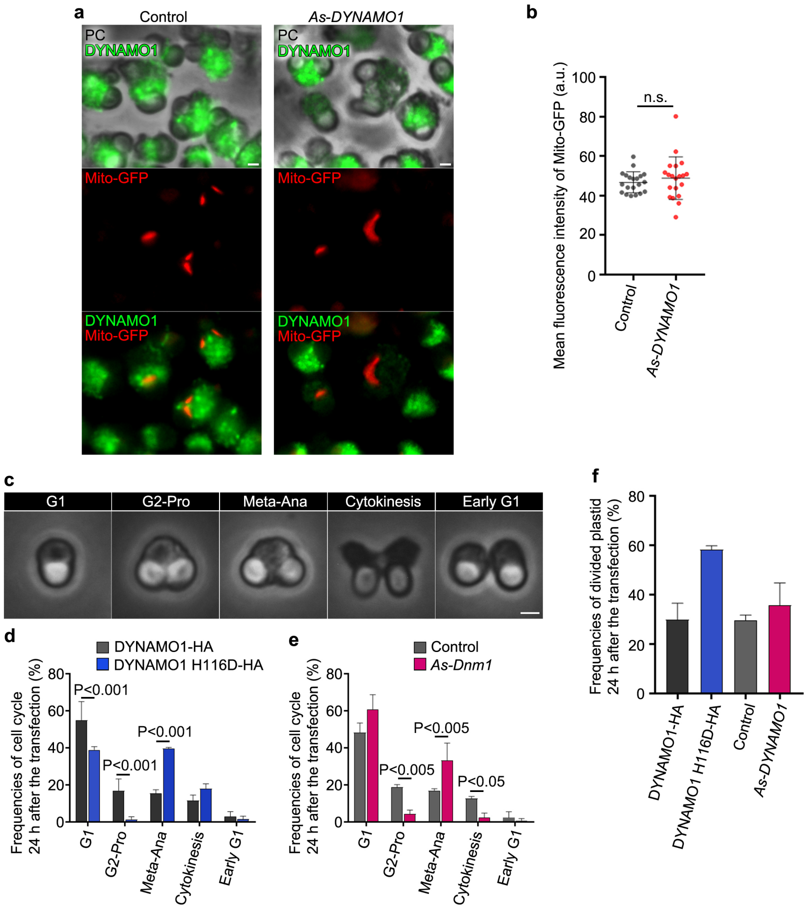

Supplementary Figure 8. Effects of DYNAMO1 inhibition. (**a** and **b**) Translation level is assessed by immunofluorescence intensity of transiently expressed mitochondrial-targeted GFP (Mito-GFP, anti-GFP antibody) in control and (*As*)-*DYNAMO1* cells. n = 2. At least 50 cells are examined in each experiment. (**c**) Phase-contrast images of typical cells in each cell cycle. (**d**) Frequencies of cell type at 24 h after transfection of DYNAMO1-HA or DYNAMO1 H116D-HA. n = 2. At least 50 cells are examined in each experiment. (**e**) Frequencies of cell type at 24 h after the transfection of control plasmid or *As-Dnm1*. n = 2. At least 50 cells are examined in each experiment. (**f**) Frequencies of divided plastid at 24 h after the transfection of DYNAMO1-HA, DYNAMO1 H116D-HA, control plasmid or *As-DYNAMO1*. Scale bars, 1 μm.

Data are means ± s.d. n.s.; not significant, P; p-value (b; unpaired *t*-test, d, e; Mann-Whitney test).

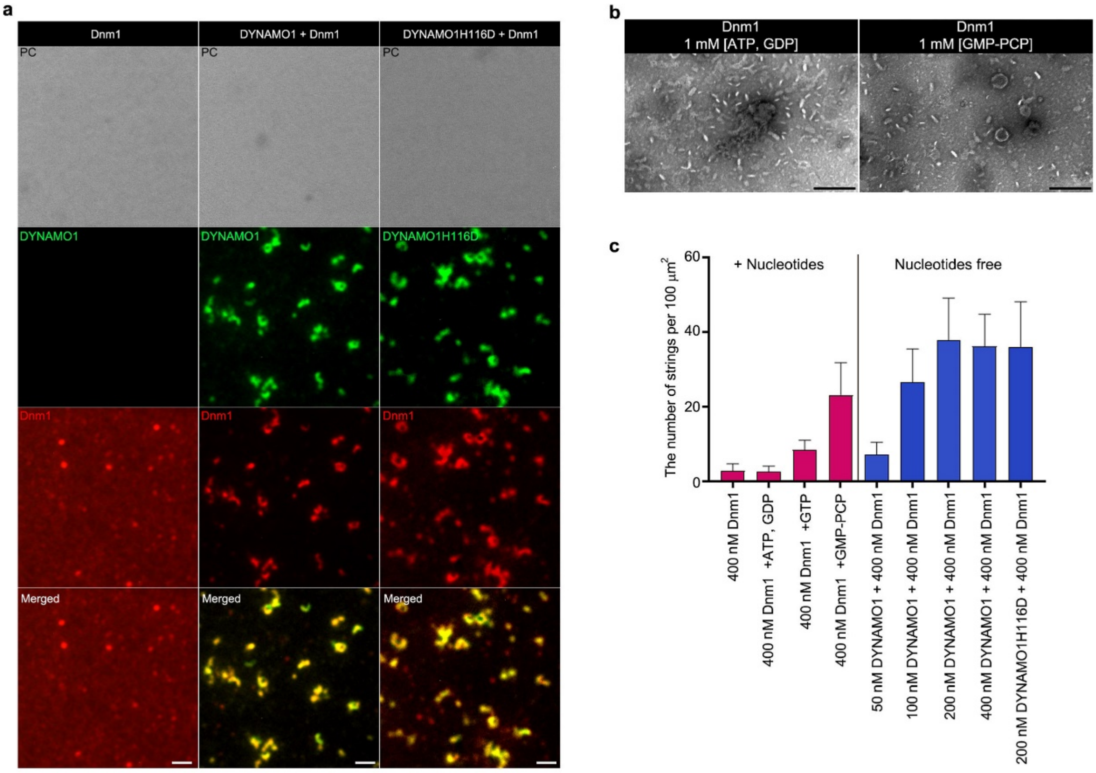

Supplementary Figure 9. Structure of DYNAMO1-Dnm1 complex. (**a**) Phase contrast and immunofluorescence microscopy images of DYNAMO1 and Dnm1 in mixtures of DYNAMO1-free recombinant Dnm1, recombinant DYNAMO1 and Dnm1, and recombinant DYNAMO1 H116D and Dnm1. Scale bars, 500 nm. (**b**) Electron microscopy images in DYNAMO1-free Dnm1 upon addition of ATP and GMP–PCP. (**c**) The number of DYNAMO1–Dnm1 or DYNAMO1-free Dnm1 strings under these various conditions. $n = 3$. Data are means ± s.d.

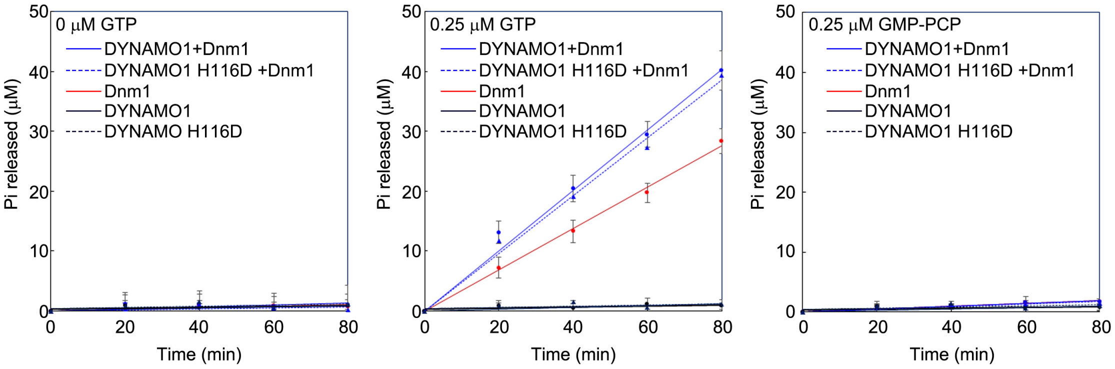

Supplementary Figure 10. GTPase activity of the DYNAMO1–Dnm1 complex. GTP hydrolysis by Dnm1 (500 nM) measured without GTP (0 μM), with 0.25 μM GTP or with 0.25 μM GMP–PCP, in the presence or absence of DYNAMO1 or DYNAMO1 H116D. *n* = 4. Data are means ± s.d.

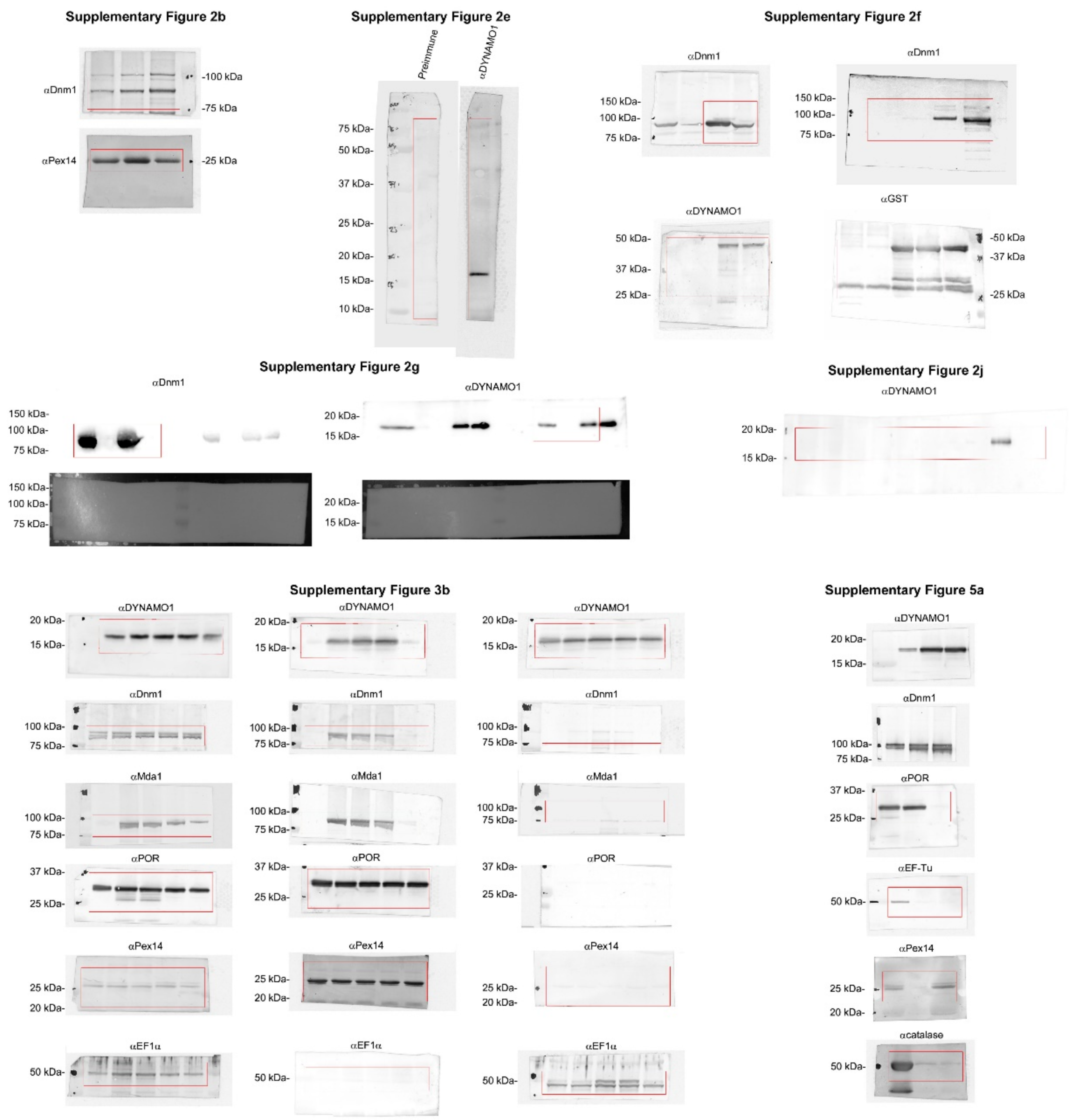

Supplementary Figure 11. All of gels used in the report are shown. Red boxes indicate cropped area shown in main Figures.

Band #	Acc. No.	Annotation	Mascot score	No. peptide	Coverage (%)	emPAI	Mass (Da)
1	CME019	dynamin-related protein involved in mitochondrial division CmDnm1/DRP3	2356	42	49.2	11.85	86639
	CMJ046	similar to kinetoplast-associated protein	125	11	12.1	0.64	86639
2	CMN145	histone H2B	329	13	73.7	53.48	13085
	CMN165	histone H3	217	8	39	7.22	15473
	CME019	dynamin-related protein involved in mitochondrial division CmDnm1/DRP3	197	9	12.1	0.55	86639
	CMJ121	hypothetical protein	149	5	25.5	2.1	18113
	CMP166	phycocyanin-associated rod linker protein	121	5	10.7	0.59	45159
	CMH254	hypothetical protein	120	4	22.1	1.66	16605
	CMS306	hypothetical protein	118	6	27.2	2.15	21563
3	CME019	dynamin-related protein involved in mitochondrial division CmDnm1/DRP3	973	32	32.3	4.56	86639
	CMJ046C	similar to kinetoplast-associated protein	256	13	15.3	0.82	89818
	CMT398C	hypothetical protein	153	7	11.9	0.5	71123
	CMP166C	phycocyanin-associated rod linker protein	124	4	8.8	0.44	45159
4	CME019C	dynamin-related protein involved in mitochondrial division CmDnm1/DRP3	464	12	15	0.84	86639
	CML063C	hypothetical protein	295	10	31.7	3.07	28426
	CMJ105C	similar to chloroplast inner membrane protein Tic22	220	11	24	2.98	40740
	CMI038C	Mg-protoporphyrin O-methyltransferase	211	8	22.9	1.61	33437
	CML110C	nucleoside-diphosphate kinase	158	6	32	3.00	17034
	CMJ259C	hypothetical protein	128	4	16	0.78	27772
	CMD062C	small GTP-binding protein of Rab family	115	2	11.2	0.37	25039
	CMG206C	similar to peroxisomal membrane protein PMP22	113	4	9	0.62	33069
	CMT028C	Mn superoxide dismutase	105	6	16.8	0.98	35119
5	CML110C	nucleoside-diphosphate kinase	3332	12	51.6	15.78	17304
	CME019C	dynamin-related protein involved in mitochondrial division CmDnm1/DRP3	430	11	16.3	0.86	86639
	CML200C	hypothetical protein, conserved	243	8	30.7	2.18	31624
	CMS306C	hypothetical protein	208	6	34	2.07	21563
	CMJ126C	hypothetical protein	115	4	13.8	1.26	24827

6	CMS306C	hypothetical protein	1094	18	70.2	30.27	21563
	CME019C	dynamin-related protein involved in mitochondrial division CmDnm1/DRP3	336	9	12.2	0.55	86639
	CMP332C	similar to fibrillin	157	7	25.8	2.03	26238
	CMI135C	single strand binding protein, SSB	139	3	10.9	0.9	25731
	CML110C	nucleoside-diphosphate kinase	127	4	25.5	1.61	17034
	CMP166C	phycocyanin-associated rod linker protein	110	5	17.9	0.59	45159
	CMR441C	hypothetical protein, conserved	110	5	16.7	1.55	22030

Supplementary Table 1. List of potential candidate proteins comprising POD machinery, identified by LC-MS/MS analysis of isolated POD machinery fraction. Band # indicates the number of band subjected to LC-MS/MS analysis as shown in Supplementary Figure 2c. Acc. No.; Accession number, No. peptide; the number of significantly unique peptide. The analysis is a single measurement.
